# Use and Reporting of Patient-Reported Outcomes in Trials of Palliative Radiotherapy: A Systematic Review

**DOI:** 10.1001/jamanetworkopen.2022.31930

**Published:** 2022-09-22

**Authors:** Alexander Fabian, Justus Domschikowski, Anne Letsch, Claudia Schmalz, Sandra Freitag-Wolf, Juergen Dunst, David Krug

**Affiliations:** 1 Department of Radiation Oncology, University Hospital Schleswig-Holstein, Kiel, Germany; 2 Department of Haematology and Oncology, University Hospital Schleswig-Holstein, Kiel, Germany; 3 Institute of Medical Informatics and Statistics, Christian-Albrechts-University Kiel, Kiel, Germany

## Abstract

**Importance:**

Approximately 50% of all patients with cancer have an indication for radiotherapy, and approximately 50% of radiotherapy is delivered with palliative intent, with the aim of alleviating symptoms. Symptoms are best assessed by patient-reported outcomes (PROs), yet their reliable interpretation requires adequate reporting in publications.

**Objective:**

To investigate the use and reporting of PROs in clinical trials of palliative radiotherapy.

**Evidence Review:**

This preregistered systematic review searched PubMed/Medline, EMBASE, and the Cochrane Center Register of Controlled Trials for clinical trials of palliative radiotherapy published from 1990 to 2020. Key eligibility criteria were palliative setting, palliative radiotherapy as treatment modality, and clinical trial design (per National Institutes of Health definition). Two authors independently assessed eligibility. Trial characteristics were extracted and standard of PRO reporting was assessed in adherence to the Consolidated Standards of Reporting Trials (CONSORT) PRO extension. The association of the year of publication with the use of PROs was assessed by logistic regression. Factors associated with higher CONSORT-PRO adherence were analyzed by multiple regression. This study is reported following the PRISMA guidelines.

**Findings:**

Among 7377 records screened, 225 published clinical trials representing 24 281 patients were eligible. Of these, 45 trials (20%) used a PRO as a primary end point and 71 trials (31%) used a PRO as a secondary end point. The most prevalent PRO measures were the Numeric Rating Scale/Visual Analogue Scale (38 trials), European Organization for the Research and Treatment of Cancer Quality of Life Questionnaire C30 (32 trials), and trial-specific unvalidated measures (25 trials). A more recent year of publication was significantly associated with a higher chance of PROs as a secondary end point (odds ratio [OR], 1.04 [95% CI, 1.00-1.07]; *P* = .03) but not as primary end point. Adherence to CONSORT-PRO was poor or moderate for most items. Mean (SD) adherence to the extension adherence score was 46.2% (19.6%) for trials with PROs as primary end point and 31.8% (19.8%) for trials with PROs as a secondary end point. PROs as a primary end point (regression coefficient, 9.755 [95% CI, 2.270-17.240]; *P* = .01), brachytherapy as radiotherapy modality (regression coefficient, 16.795 [95% CI, 5.840-27.751]; *P* = .003), and larger sample size (regression coefficient, 0.028 [95% CI, 0.006-0.049]; *P* = .01) were significantly associated with better PRO reporting per extension adherence score.

**Conclusions and Relevance:**

In this systematic review of palliative radiotherapy trials, the use and reporting of PROs had room for improvement for future trials, preferably with PROs as a primary end point.

## Key Points

**Question:**

How commonly are patient-reported outcomes (PROs) used and how well are they reported in clinical trials of palliative radiotherapy?

**Findings:**

In this systematic review that included 225 published clinical trials representing 24 281 patients, only 20% of trials used PROs as a primary end point and 31% as a secondary end point. The reporting of PROs was poor or moderate for most items of the Consolidated Standards of Reporting Trials PRO extension, while the use of a PRO as a primary end point was significantly associated with better reporting.

**Meaning:**

These findings suggest that the current use and reporting of PROs has room for improvement in future trials of palliative radiotherapy.

## Teaser

This systematic review investigates use and reporting of patient-reported outcomes in clinical trials of palliative radiotherapy.

## Introduction

At least 50% of all patients with cancer have an indication for radiotherapy.^1^ Additionally, approximately 50% of radiotherapies for patients with cancer are delivered with a palliative intent.^2^ Palliative radiotherapy mainly aims to comfort patients and to stabilize or improve symptoms and quality of life.^3^ Symptoms and quality of life are best captured by asking the patient themself, without interpretation by proxy, ie, by patient-reported outcomes (PROs).^4^ PROs are particularly important when survival benefits of an intervention are marginal or not expected.^5^ A 2021 cohort study^6^ underlined this aspect, as it reported a 30-day mortality rate of 24% in 518 patients after palliative radiotherapy. Consequently, guidelines encourage the use of PROs in clinical trials of palliative radiotherapy, such as for bone metastasis.^7^ The implementation of PROs is feasible in caring for patients treated with palliative intent.^8^ Yet it is unclear if the use of PROs in clinical trials of palliative radiotherapy has increased over time, as has been reported for other types of clinical studies.^9^

However, evidence from PROs may be undermined by methodological issues, including but not limited to the use of unvalidated PRO measures (PROMs). For example, analyses have shown that quality standards in the use of PROs in brain or head and neck cancer trials are challenged by suboptimal reporting.^10,11^ Therefore, the Consolidated Standards of Reporting Trials (CONSORT) Statement of 2010 was supplemented in 2013 by the CONSORT-PRO extension,^12^ which addressed how to best report PROs in trial publications. Since then, specific trial characteristics have been associated with favorable PRO reporting, such as PROs being the primary end point or a more recent date of publication.^13,14^

To our knowledge, there is no systematic overview on the use and reporting of PROs in trials of palliative radiotherapy, despite the relevance of PROs in this setting. This overview could aid to determine the status quo, depict trends over time, reveal favorable trial characteristics, and highlight areas of improvement for future palliative radiotherapy trials concerning the use and reporting of PROs.

Therefore, we performed a systematic review of clinical trials of palliative radiotherapy. Our primary objectives were to characterize the use of PROs in trials of palliative radiotherapy and to assess the standard of reporting of PROs per adherence to the CONSORT-PRO extension.^12^ Adherence to the CONSORT-PRO extension^12^ was to be investigated using predefined trial characteristics. A secondary objective was to describe registered, ongoing trials of palliative radiotherapy and their use of PROs.

## Methods

This study is a systematic review as meta-research analysis because we analyzed methodological patterns in trials rather than effect sizes of outcomes.^15^ We followed the Preferred Reporting Items for Systematic Reviews and Meta-analyses (PRISMA) reporting guideline wherever appropriate in the literature search and data extraction. A study protocol was preregistered online^16^ and is available in the eAppendix 1 in the Supplement. In addition to the aforementioned objectives, a detailed analysis of end points in trials of palliative radiotherapy in general was another objective per protocol, which are reported elsewhere.^17^

### Systematic Literature Search

We searched the databases PubMed/Medline, EMBASE, and the Cochrane Center Register of Controlled Trials (CENTRAL) for studies published between 1990 and 2020. This period covered 3 decades and started at 1990 because more PROMs became available in the early 1990s.^18^ ClinicalTrials.gov was searched for registered, ongoing studies. The search was conducted in March 2021 and was supported by a professional librarian. The population, intervention, comparison, and outcome elements and exact search strategy are detailed in the protocol (eAppendix 1 in the Supplement) and included palliative setting (population), radiotherapy (intervention), and clinical trials (study type). We used the Covidence systematic review software (Veritas Health Innovation) for record management. Two authors (A.F. and J. Domschikowski) independently screened records for eligibility at the title and abstract stage and at the full-text stage. Disagreements were settled by discussion among coauthors.

### Eligibility Criteria

Inclusion criteria for published trials and registrations were including patients with cancer in a palliative treatment setting (as defined by the authors of the trial), a clinical trial per definition of the National Institutes of Health (ie, “a research study in which one or more human subjects are prospectively assigned to one or more interventions… to evaluate the effects of those interventions on health-related biomedical or behavioral outcomes”),^19^ with palliative radiotherapy as an integral part of the study or control treatment, and including at least 1 clinical outcome. Exclusion criteria were an oligometastatic setting (if it was a single-group trial of locally curative or ablative therapy), assessing treatment with curative intent, assessing supportive medication or validation of outcome assessment tools as focus of the study, in abstract format only, and published a language other than English.

### Variables and Data Extraction

We used controlled vocabulary with exact definitions of relevant terms for data extraction, as predefined in the protocol (eAppendix 1 in the Supplement). These included the definition for PRO from Calvert and colleagues^20^ as “an outcome reported directly by patients themselves and not interpreted by an observer; PROs may include patient assessments of health status, quality of life, or symptoms.” Predefined extracted variables included general trial characteristics. PROs were categorized as primary end point if the trial clearly stated them as primary end point. In all other cases, PROs were categorized as secondary end points, if they were used. We also extracted CONSORT-PRO scores for published trials with PROs.^21^ CONSORT-PRO scores were rated in terms of 2 different complementary scores, as described elsewhere.^21^ In brief, the extension adherence score included all PRO-related items that were newly added in the CONSORT-PRO extension.^12^ The score includes 5 items, of which 2 items are further subdivided, resulting in 8 items to analyze. In addition to these items, the total CONSORT-PRO adherence score also included pre-existing CONSORT items relevant to the reporting of PROs, resulting in 19 subitems. As in the scoring method used, item 4a (PRO used in eligibility or stratification) was not evaluated because this would have required access to trial protocols. Adherence to specific CONSORT-PRO items across trials was ranked as good (≥80%), moderate (50%-79%), or poor (≤49%), as described elsewhere.^21^

Data extraction of eligible records was performed via predefined data extraction forms. Multiple publications of the same trial were merged to 1 record. Two authors (A.F. and J. Domschikowski) independently extracted general characteristics of 20 records for published trials and 10 records for trial registrations, resulting in an interrater agreement of 96% among published trials and 94% among trial registrations. Of the 20 published trials, 8 included at least 1 PRO. For these trials, 2 authors (A.F. and J. Domschikowski) independently extracted data on CONSORT-PRO scores,^21^ resulting in an interrater agreement of 85%. To confirm this rate, an additional 5 trials were independently assessed by 2 authors (A.F. and J. Domschikowski) for CONSORT-PRO scores, resulting in an interrater agreement of 83%, which was considered adequate and comparable with a similar analysis by Kyte et al.^22^ Data of all remaining records were extracted either by A.F. or J. Domschikowski and discussed in case of uncertainties.

### Statistical Analysis

We used descriptive statistics to characterize data. Using logistic regression, we investigated the association between the year of publication and the use of PROs. We also used logistic regression to analyze the association between year of publication and use of an unvalidated PROM. We converted raw item adherence numbers of the CONSORT-PRO scores to percentages for further analysis, as the applicable item number was dependent on a PRO being a primary or secondary end point. The maximum raw extension adherence score was 7 for every trial. The maximum raw total CONSORT-PRO adherence score was 15 for trials with a PRO as a primary end point and 14 for trials with a PRO as a secondary end point.^21^ Using 2 different multiple regression models, we assessed associations of the extension adherence score or the total CONSORT-PRO adherence score as the dependent variable and predefined trial characteristics as independent variables. Predefined trial characteristics included use of PROs as a primary vs secondary end point, study design (randomized vs nonrandomized trial and single-center vs multicenter trial), sample size, modality of radiotherapy, and year of publication. These characteristics were selected based on previous publications, the ability to robustly assess these variables, and a hypothesized need to include potentially confounding factors, such as trial design.^11,13,23^

We used the software JASP software version 0.16 (JASP Team) and SPSS version 27 (IBM Corp) for statistical analyses. *P* values were 2-sided, and statistical significance was set at *P* < .05. Data were analyzed from October 2021 to March 2022.

## Results

**Figure 1.**
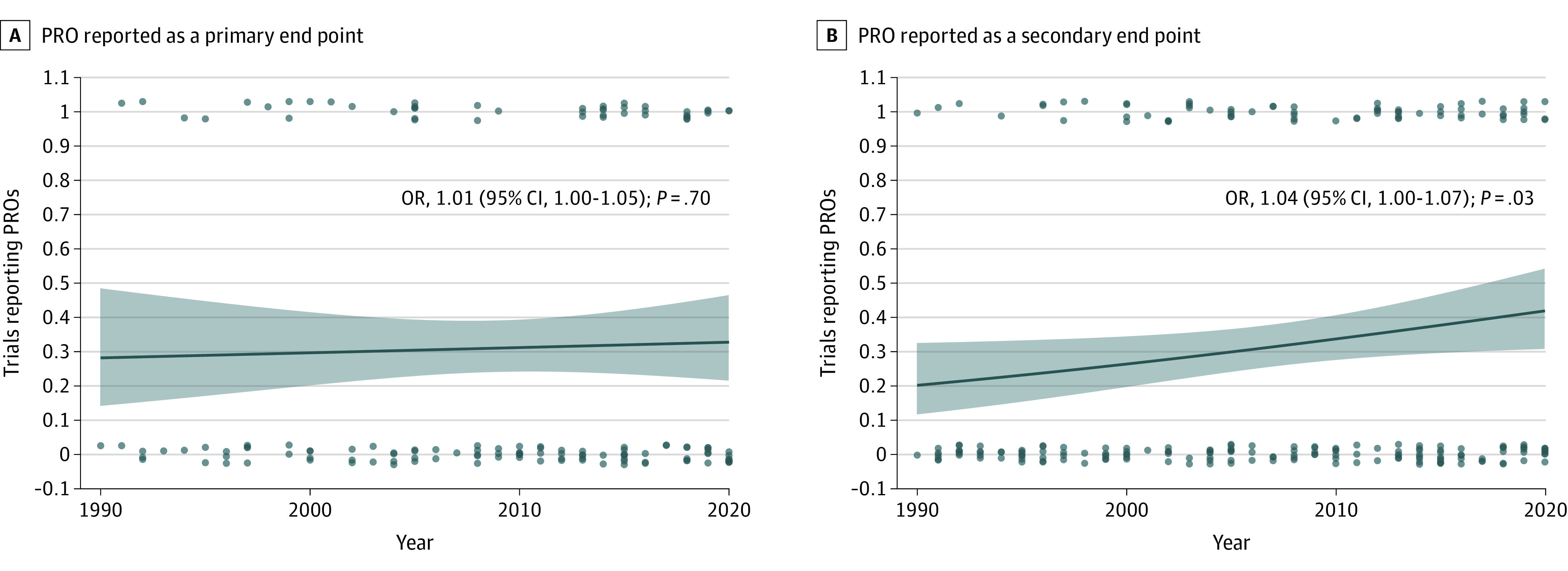
Patient-Reported Outcomes (PRO) in Published Trials of Palliative Radiotherapy Over Time Logistic regression analysis of the use of a PRO end point (1 indicates yes; 0, no) as dependent variable and year of publication as independent variable. A, Use of PROs as primary end points among 145 trials clearly stating their endpoint. B, Use of PROs as secondary end points among all 225 assessed trials (n = 225). Dots indicate data points; lines, mean; shading, 95% CI.

The literature search resulted in 7377 records to screen (eFigure 1 in the Supplement). A total of 225 published trials, representing 24 281 patients, and 67 trial registrations met the general eligibility criteria (eAppendix 2 in the Supplement). Among the published trials, 116 trials^24,25,26,27,28,29,30,31,32,33,34,35,36,37,38,39,40,41,42,43,44,45,46,47,48,49,50,51,52,53,54,55,56,57,58,59,60,61,62,63,64,65,66,67,68,69,70,71,72,73,74,75,76,77,78,79,80,81,82,83,84,85,86,87,88,89,90,91,92,93,94,95,96,97,98,99,100,101,102,103,104,105,106,107,108,109,110,111,112,113,114,115,116,117,118,119,120,121,122,123,124,125,126,127,128,129,130,131,132,133,134,135,136,137,138,139^ (52%) used PROs. PROs were primary end points in 45 trials^20,26,28,30,34,35,36,37,42,48,53,56,57,58,61,62,63,66,70,74,75,77,79,83,85,93,94,97,103,108,109,110,113,115,117,118,124,125,127,128,130,133,134,137^ (20% of all published trials; 31% of 145 trials clearly stating their primary end point; 39% of 116 trials including a PRO) and secondary end points in 71 trials^24,25,27,29,31,32,33,38,40,41,43,44,45,46,47,49,50,51,52,54,55,59,60,64,65,67,68,69,71,72,73,76,78,80,81,82,84,86,87,88,89,90,91,92,95,96,98,99,100,101,102,104,105,106,107,111,112,114,116,119,121,122,126,129,131,132,135,136,138,139^ (31% of all published trials; 61% of trials including a PRO). In published trials, a more recent year of trial publication was associated with a more frequent use of a PRO as a secondary (odds ratio [OR], 1.04 [95% CI, 1.00-1.07]; *P* = .03), but not as a primary (OR, 1.01 [95% CI, 0.97-1.05]; *P* = .7) end point, per logistic regression (Figure 1). There were no statistically significant trends over time in the use of PROs between randomized and nonrandomized trials (eFigure 2 in the Supplement).

**Figure 2.**
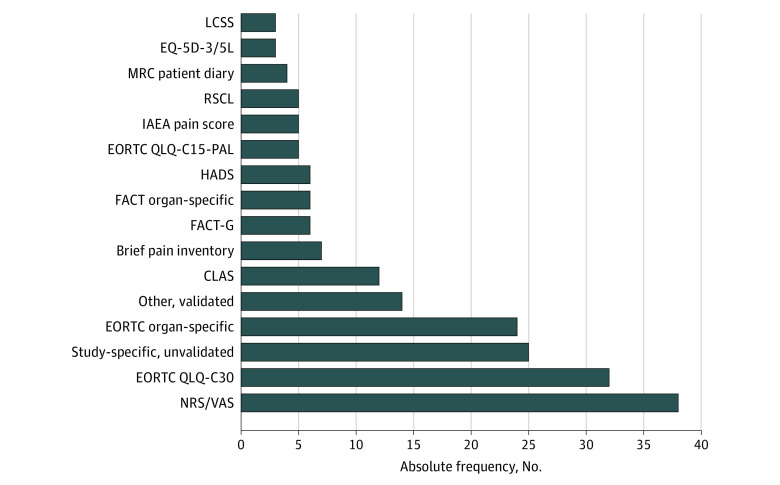
Patient-Reported Outcome Measures (PROMs) Used in Published Trials of Palliative Radiotherapy (N = 116) Multiple measures could be used per trial. CLAS indicates Cancer Linear Analogue Scale; EORTC, European Organization for Research and Treatment of Cancer; FACT, Functional Assessment of Cancer Therapy; HADS, Hospital Anxiety and Depression Scale; IAEA, International Atomic Energy Agency; LCSS, Lung Cancer Symptom Scale; MRC, Medical Research Council; NRS/VAS, numeric rating scale/visual analogue scale; RSCL, Rotterdam Symptom Checklist.

The most prevalent characteristics of trials using PROs were single-center, randomized, no industry funding, Europe-based, and use of external beam radiotherapy as the radiotherapy modality (Table 1). Median (IQR) sample size was 98 (41-230) patients in trials with a PRO as a primary end point, and 50 (31-132) patients in trials with a PRO as a secondary end point. Metastases and thoracic primaries were the most common treated sites (eFigure 3 in the Supplement), including in 37 published trials^26,28,30,34,35,36,37,42,48,53,57,61,63,70,74,75,77,79,83,85,93,94,103,108,109,110,113,115,117,118,123,124,125,126,128,133,137^ (82%) with a PRO as a primary end point and in 50 published trials^27,29,31,32,33,38,39,40,41,43,44,46,51,54,55,65,67,68,69,71,72,73,76,78,80,81,84,86,87,88,90,91,92,95,96,99,100,101,102,105,107,114,116,121,122,126,135,136,138,139^ (70%) with a PRO as a secondary end point. The most prevalent PROMs were the Numeric Rating Scale or Visual Analogue Scale (38 trials^28,29,30,35,37,40,42,43,44,45,56,57,59,60,61,63,67,68,69,72,73,74,80,81,82,87,97,101,103,111,112,113,118,122,123,124,138,140^), European Organization for the Research and Treatment of Cancer Quality of Life Questionnaire C30 (32 trials^24,25,26,33,34,36,41,48,49,51,53,64,73,76,78,83,84,85,88,89,90,93,98,107,111,112,120,126,127,129,130,134^), and trial-specific unvalidated measures (25 trials^27,31,36,39,47,50,51,52,70,79,99,101,102,108,109,114,116,117,119,132,133,135,136,137^) (Figure 2). A more recent year of publication was significantly associated with a less frequent use of trial-specific unvalidated PROMs (OR, 0.89 [95% CI, 0.84-0.95]; *P* < .001) per logistic regression.

**Table 1.** Characteristics of Eligible Published Trials Including PROs as Primary or Secondary End Points

Characteristic	PRO reported as end point, No. (%)^a^	
	Primary (n = 45)	Secondary (n = 71)
Study design		
Multicenter	20 (44)	24 (34)
Phase		
I	1 (2)	6 (6)
I or II	73 (3)	9 (6)
II	7 (16)	11 (16)
III	11 (24)	11 (16)
IV	0	1 (1)
Exploratory	2 (4)	6 (9)
Not stated	21 (47)	32 (45)
Randomized	28 (62)	36 (51)
Funding by industry	3 (7)	5 (7)
Location		
Europe	23 (51)	34 (48)
Asia	7 (16)	17 (24)
North America	5 (11)	6 (9)
Africa	3 (7)	4 (6)
Oceania	2 (4)	6 (49)
Multiple	5 (11)	6 (9)
Radiotherapy modality		
EBRT	36 (80)	62 (87)
BT	3 (7)	6 (9)
EBRT with BT	2 (4)	2 (3)
SRS/SBRT	3 (7)	1 (1)
Other	1 (2)	0
Concurrent systemic therapy	4 (9)	11 (16)
Chemotherapy	2 (4)	7 (10)
Targeted therapy	1 (2)	2 (3)
Immunotherapy	0	0
Other	1 (2)	2(3)

Abbreviations: BT, brachytherapy; EBRT, external beam radiotherapy; PRO, patient-reported outcome; SRS/SBRT, radiosurgery/stereotactic body radiotherapy.

^a^ Percentages may not add up to 100 due to rounding error, missing information in published studies, or multiple values per study.

The standard of PRO reporting in published trials of palliative radiotherapy per adherence to all CONSORT-PRO items is shown in Table 2. No trial cited the CONSORT-PRO extension. In total, trials with a PRO as a primary end point scored good on 2 items (10%), moderate on 9 items (45%), and poor on 9 items (45%). Trials with a PRO as a secondary end point scored good on 1 item (5%), moderate on 4 items (21%), and poor on 14 items (74%). Good adherence was present for the item 2a (ie, rationale for including PRO end point) regardless of the PRO being the primary or secondary end point. In trials with a PRO as a primary end point, good adherence was also present for item 17ai (ie, PRO results reported for hypothesized domains and time point or for each domain if no PRO hypothesis provided). Poorest adherence was seen for items P2bi (ie, PRO hypothesis present) and P2bii (ie, PRO domains in hypothesis). Mean (SD) trial adherence to the extension adherence score was 46.2% (19.6%) in trials with a PRO as a primary end point and 31.8% (19.8%) in trials with a PRO as a secondary end point. Mean (SD) total CONSORT-PRO adherence score was 54.0% (17.4%) in trials with a PRO as a end point and 36.7% (17.9%) in trials with a PRO as secondary end point.

**Table 2.** CONSORT-PRO Item Adherence Scores in Studies of Palliative Radiotherapy With PRO as Primary or Secondary End Points

CONSORT-PRO item	Trials with PRO as primary end point (n = 45)		Trials with PRO as secondary end point (n = 70)^a^	
	No. (%)	Adherence^b^	No. (%)	Adherence^b^
Abstract: P1b: PRO as primary or secondary end point				
P1b: completely addressed	22 (49)	Poor	10 (14)	Poor
P1b: partially addressed	13 (29)	Poor	30 (43)	Poor
Introduction				
P2a: rationale for including PRO end point	40 (89)	Good	56 (80)	Good
P2bi: PRO hypothesis present	3 (7)	Poor	4 (6)	Poor
P2bii: PRO domains in hypothesis	3 (7)	Poor	2 (3)	Poor
Methods				
P6ai: evidence of PRO instrument validity cited	31 (69)	Moderate	43 (61)	Moderate
P6aii: statement of the person completing the PRO	31 (69)	Moderate	38 (54)	Moderate
P6aiii: mode of administration (eg, paper, e-PRO)	9 (20)	Poor	6 (9)	Poor
P7a: how sample size was determined (only if PRO is a primary end point)	24 (53)	Moderate	0	NA
P12a: statistical approach for dealing with missing data	10 (22)	Poor	4 (6)	Poor
Results				
P13ai: reports No. of questionnaires available at baseline	30 (67)	Moderate	26 (37)	Poor
P13aii: reports No. of questionnaires available at subsequent time points	29 (64)	Moderate	26 (37)	Poor
P15: Demographics table includes baseline PRO	16 (36)	Poor	11 (16)	Poor
P16: No. of patients included in each PRO analysis	28 (62)	Moderate	31 (44)	Poor
P17ai: PRO results reported for the hypothesized domains and time point specified in the hypothesis or reported for each domain of the PRO questionnaire if no PRO hypothesis provided	31 (84)	Good^c^	29 (55)	Moderate^c^
P17aii: results include CI, effect size, or some other estimate of precision	24 (53)	Moderate	27 (39)	Poor
P18: results of subgroup, adjusted, or exploratory analyses	17 (38)	Poor	5 (7)	Poor
Discussion				
P20: PRO-specific study limitations provided	22 (49)	Poor	25 (36)	Poor
P21: implications of PRO results for generalizability and clinical practice	31 (69)	Moderate	34 (49)	Poor
P22: PROs interpreted in relation to clinical outcomes	35 (78)	Moderate	44 (63)	Moderate
Total items				
With good adherence	2 (10)	NA	1 (5)	NA
With moderate adherence	9 (45)	NA	4 (21)	NA
With poor adherence	9 (45)	NA	14 (74)	NA

Abbreviations: CONSORT, Consolidated Standards of Reporting Trials; NA, not applicable; PRO, patient-reported outcome.

^a^ One trial stated a PRO was an end point in its primary manuscript, but PRO results were not yet reported at the time of our analysis. Therefore, 70 trials were assessed for adherence to CONSORT-PRO.

^b^ Adherence rating within each group of items was defined as good, 80% or greater; moderate, 50% to 79%; and poor, 49% or less.

^c^ Item P17ai was not assessed for 17 publications with only 1 PRO domain.

Next, we assessed associations of predefined characteristics of published trials and the extension adherence score or total CONSORT-PRO adherence score using 2 separate multiple regression models (eTable 1 in the Supplement). Higher adherence to the extension adherence score was significantly associated with a PRO being a primary end point (β = 9.755 [95% CI, 2.270-17.240]; *P* = .01), brachytherapy as modality of radiotherapy (β = 16.795 [95% CI, 5.840-27.751]; *P* = .003), and larger sample size (β = 0.028 [95% CI, 0.006-0.049]; *P* = .01) (Table 3). Higher adherence to the total CONSORT-PRO adherence score was also significantly associated with a PRO being a primary end point (β = 12.694 [95% CI, 6.081-19.307]; *P* < .001), brachytherapy as modality of radiotherapy (β = 14.115 [95% CI, 4.437-23.794]; *P* = .005), and higher patient number (β = 0.033 [95% CI, 0.013-0.052]; *P* = .001) (Table 3). In addition, multicenter study design was associated with higher total CONSORT-PRO adherence score (β = 7.185 [95% CI, 0.175-14.195]; *P* = .045) (Table 3). To assess an association of the publication date of the CONSORT-PRO extension with PRO reporting, we conducted a post hoc analysis adapting year of publication to date of publication later than 2013. There was no statistically significant association of date of publication after 2013 with PRO reporting per any of the CONSORT-PRO scores (eTable 2 and eTable 3 in the Supplement).

**Table 3.** Factors Associated With the Degree of PRO Reporting in Trials of Palliative Radiotherapy

Independent variable	Dependent variable			
	Extension Adherence score		Total CONSORT-PRO Adherence score	
	β (95% CI)	*P* value	β (95% CI)	*P* value
Constant	102.75 (−815.3 to 1020.6)	.83	−495.74 (−1306.7 to 315.2)	.23
Year of publication	−0.047 (−0.504 to 0.409)	.84	0.255 (−0.148 to 0.658)	.21
PRO as primary end point	9.755 (2.270 to 17.240)	.01	12.694 (6.081 to 19.307)	<.001
Randomization	−0.013 (−8.144 to 8.119)	>.99	−1.246 (−8.430 to 5.938)	.73
Multicenter trial	7.328 (−0.606 to 15.262)	.07	7.185 (0.175 to 14.195)	.045
Modality of radiotherapy, BT	16.795 (5.840 to 27.751)	.003	14.115 (4.437 to 23.794)	.005
Patient No.	0.028 (0.006 to 0.049)	.01	0.033 (0.013 to 0.052)	.001

Abbreviations: β, regression coefficient; BT, brachytherapy; CONSORT, Consolidated Standards of Reporting Trials; PRO, patient-reported outcome.

Among 67 ongoing, registered trials of palliative radiotherapy, 37 registrations^140,141,142,143,144,145,146,147,148,149,150,151,152,153,154,155,156,157,158,159,160,161,162,163,164,165,166,167,168,169,170,171,172,173,174,175,176^ (55%) use a PRO, including 20 trials^142,143,145,146,147,148,149,150,151,152,153,155,158,162,164,165,166,169,175,176^ (30%) with a PRO as a primary end point and 17 trials^140,141,144,154,156,157,159,160,161,163,167,168,170,171,172,173,174^ (25%) with a PRO as a secondary end point. Of note, no trial uses brachytherapy alone, and only 1 trial^164^ uses brachytherapy in combination with external beam radiotherapy (eTable 4 in the Supplement). The median IQR planned sample size is 65 (36-108) patients. Metastases, thoracic primaries, and abdominal primaries are the most common planned treatment sites (eFigure 4 in the Supplement).

## Discussion

In this systematic review of PROs in trials of palliative radiotherapy, we found that approximately half of all eligible published trials used PROs and 20% of these used a PRO as a primary end point. No trial cited the CONSORT-PRO extension. The standard of reporting PROs was dominated by moderate or poor adherence to CONSORT-PRO items. Adherence was significantly higher in trials with a PRO as a primary end point, use of brachytherapy as radiotherapy modality, and larger sample sizes.

The overall use of PROs in trials of palliative radiotherapy of approximately 50% was modest and concentrated on few treated sites, namely metastases and thoracic primaries. This finding is in line with systematic reviews of palliative radiotherapy for head and neck, rectal, or prostate cancer that identified few trials with PROs.^177,178,179^ Other areas of cancer trials may use PROs to an even lesser extent, as illustrated by a systematic review by Riedl et al assessing childhood cancer trial registrations, which found that only 8.2% of trials used PROs.^180^ However, the use of PROs in trials of palliative radiotherapy is still suboptimal, even compared with other radiotherapy trials. A recent analysis of radiotherapy trials within the National Cancer Institute National Clinical Trials Network reported that 56% of these trials used PROs, compared with 52% in our analysis.^181^ Furthermore, a meta-research analysis of trial registrations in palliative care reported that 61% of interventional trials used a PRO as a primary end point, compared with 20% for published trials and 30% for trial registrations in our analysis.^182^ However, it should be considered a positive development that the use of unvalidated PROMs in trials of palliative radiotherapy has significantly declined over the last 30 years, as shown by our analysis. Taken together, there is room for improvement concerning the rate of PROs in trials of palliative radiotherapy, given their importance in this setting. This view is also supported by a topical review by Oldenburger et al^183^ of PROMs in palliative radiotherapy, which narratively flagged initial concerns of reporting of PROs.

The standard of PRO reporting is essential for a reliable interpretation of trial results and therefore has been investigated in several settings of cancer trials. For example, Mercieca-Bebber and colleagues^11^ identified poorly reported aspects of data collection, analysis, and generalizability of PROs in randomized clinical trials of head and neck or thyroid cancer. Furthermore, a study of phase III trials of systemic cancer therapies highlighted that methodological items of the CONSORT-PRO extension in particular are often poorly reported.^184^ Similarly, a more recent study of PRO in randomized clinical trials of therapies for hematological malignant neoplasms still noted methodological issues, such as the reporting of statistical approaches to missing data.^23^ In line with our results, these analyses showed that the CONSORT-PRO item P2b (ie, PRO hypothesis) ranked among the most poorly reported items.^9,13,184^ Several studies also investigated associations of trial characteristics with the standard of PRO reporting. Use of a PRO as a primary end point, compared with as a secondary end point, was frequently associated with better reporting.^11,13,14,23^ Larger sample size was also described as factor associated with better reporting.^14^ However, whether the reporting of PROs has improved in trials over time is not entirely clear based on the literature available so far.^9,14^

The extensive literature on reporting of PROs in different settings of cancer trials fits well with our results. These results reflect several poorly reported aspects of PROs in trials of palliative radiotherapy, which were not limited to only methodological CONSORT-PRO items. A clear presentation of these areas of improvement, as systematically evidenced by our analysis, is paramount in the context of palliative radiotherapy and its aims to improve symptoms and quality of life. For this reason, it is encouraging to see that some trials of palliative radiotherapy reported PROs better than others, such as trials with a PRO as a primary end point. Finally, not a single trial cited the CONSORT-PRO extension, which reflects the low rate (1.4%) of clinical trials citing the CONSORT statement.^185^ Authors and journals can only be encouraged to take advantage of these guidelines, as citing the CONSORT-PRO extension has been associated with better PRO reporting.^21^

### Limitations

Our study has some limitations. We used a previously reported method to score the adherence of a published trial to the CONSORT-PRO extension as measure for standard of reporting of PRO.^21^ This method was also recommended by a 2022 review of reviews by Mercieca-Bebber et al.^186^ Although the CONSORT-PRO extension is specifically for randomized clinical trials, we also applied it to nonrandomized trials, since its items are equally important to nonrandomized interventional trials. To our knowledge, there was no guideline, let alone scoring method, for the reporting of PROs for nonrandomized trials at the conception of our protocol. Furthermore, the internal validity of the extracted data on CONSORT-PRO adherence may be challenged by the fact that most trials were assessed by 1 of 2 investigators only, although we ensured an adequate rate of interrater agreement.

## Conclusions

This systematic review of PROs in trials of palliative radiotherapy found PROs in approximately half of all trials, with suboptimal PRO reporting overall. These findings suggest potential areas of improvement for future trials. Encouragingly, some trials had better reporting of PROs, such as trials with a PRO as a primary end point. Finally, more well-reported trials of palliative radiotherapy with PROs as primary end points are needed, given the considerable number of affected patients and the importance of PROs.

## Notes

Supplement. **eFigure 1.** PRISMA Flowchart **eFigure 2.** Patient-Reported Outcomes (PRO) in Published Trials of Palliative Radiotherapy Over Time Separately Analyzed for Randomized and Nonrandomized Trials **eFigure 3.** Treated Sites in Published Trials of Palliative Radiotherapy Including Patient-Reported Outcomes as Primary (n=45) or Secondary End Points (n=71) **eTable 1.** Summary Parameters of the Multiple Regression Models **eTable 2.** Factors Associated With of the Degree of Patient-Reported Outcome Reporting in Trials of Palliative Radiotherapy **eTable 3.** Summary Parameters of the Multiple Regression Models **eTable 4.** Characteristics of Ongoing Trials Registered on ClinicalTrials.gov With Patient-Reported Outcomes as End Points **eFigure 4.** Treated Sites in Ongoing, Registered Trials of Palliative Radiotherapy Including Patient-Reported Outcomes **eAppendix 1.** Study Protocol **eAppendix 2.** References of Included Published Trials of Palliative Radiotherapy and Trial Registrations
