# Supplementary material for: Use and Reporting of Patient-Reported Outcomes in Trials of Palliative Radiotherapy: A Systematic Review
Source: JAMA Netw Open. 2022 Sep 22;5(9):e2231930. doi: 10.1001/jamanetworkopen.2022.31930 (PMC9500555; doi:10.1001/jamanetworkopen.2022.31930)
Supplement: Supplement. — eFigure 1. PRISMA Flowchart eFigure 2. Patient-Reported Outcomes (PRO) in Published Trials of Palliative Radiotherapy Over Time Separately Analyzed for Randomized and Nonrandomized Trials eFigure 3. Treated Sites in Published Trials of Palliative Radiotherapy Including Patient-Reported Outcomes as Primary (n=45) or Secondary End Points (n=71) eTable 1. Summary Parameters of the Multiple Regression Models eTable 2. Factors Associated With of the Degree of Patient-Reported Outcome Reporting in Trials of Palliative Radiotherapy eTable 3. Summary Parameters of the Multiple Regression Models eTable 4. Characteristics of Ongoing Trials Registered on ClinicalTrials.gov With Patient-Reported Outcomes as End Points eFigure 4. Treated Sites in Ongoing, Registered Trials of Palliative Radiotherapy Including Patient-Reported Outcomes eAppendix 1. Study Protocol eAppendix 2. References of Included Published Trials of Palliative Radiotherapy and Trial Registrations [file jamanetwopen-e2231930-s001.pdf]

Supplemental Online Content

Fabian A, Domschikowski J, Letsch A, et al. Use and reporting of patient-reported outcomes in trials of palliative radiotherapy: a systematic review. *JAMA Netw Open*. 2022;5(9):e2231930. doi:10.1001/jamanetworkopen.2022.31930

eFigure 1. PRISMA Flowchart
eFigure 2. Patient-Reported Outcomes (PRO) in Published Trials of Palliative Radiotherapy Over Time Separately Analyzed for Randomized and Nonrandomized Trials
eFigure 3. Treated Sites in Published Trials of Palliative Radiotherapy Including Patient-Reported Outcomes as Primary (n=45) or Secondary End Points (n=71)
eTable 1. Summary Parameters of the Multiple Regression Models
eTable 2. Factors Associated With the Degree of Patient-Reported Outcome Reporting in Trials of Palliative Radiotherapy
eTable 3. Summary Parameters of the Multiple Regression Models
eTable 4. Characteristics of Ongoing Trials Registered on ClinicalTrials.gov With Patient-Reported Outcomes as End Points
eFigure 4. Treated Sites in Ongoing, Registered Trials of Palliative Radiotherapy Including Patient-Reported Outcomes
eAppendix 1. Study Protocol
eAppendix 2. References of Included Published Trials of Palliative Radiotherapy and Trial Registrations

This supplemental material has been provided by the authors to give readers additional information about their work.

© 2022 Fabian A et al. *JAMA Network Open.*

eFigure 1. PRISMA Flowchart

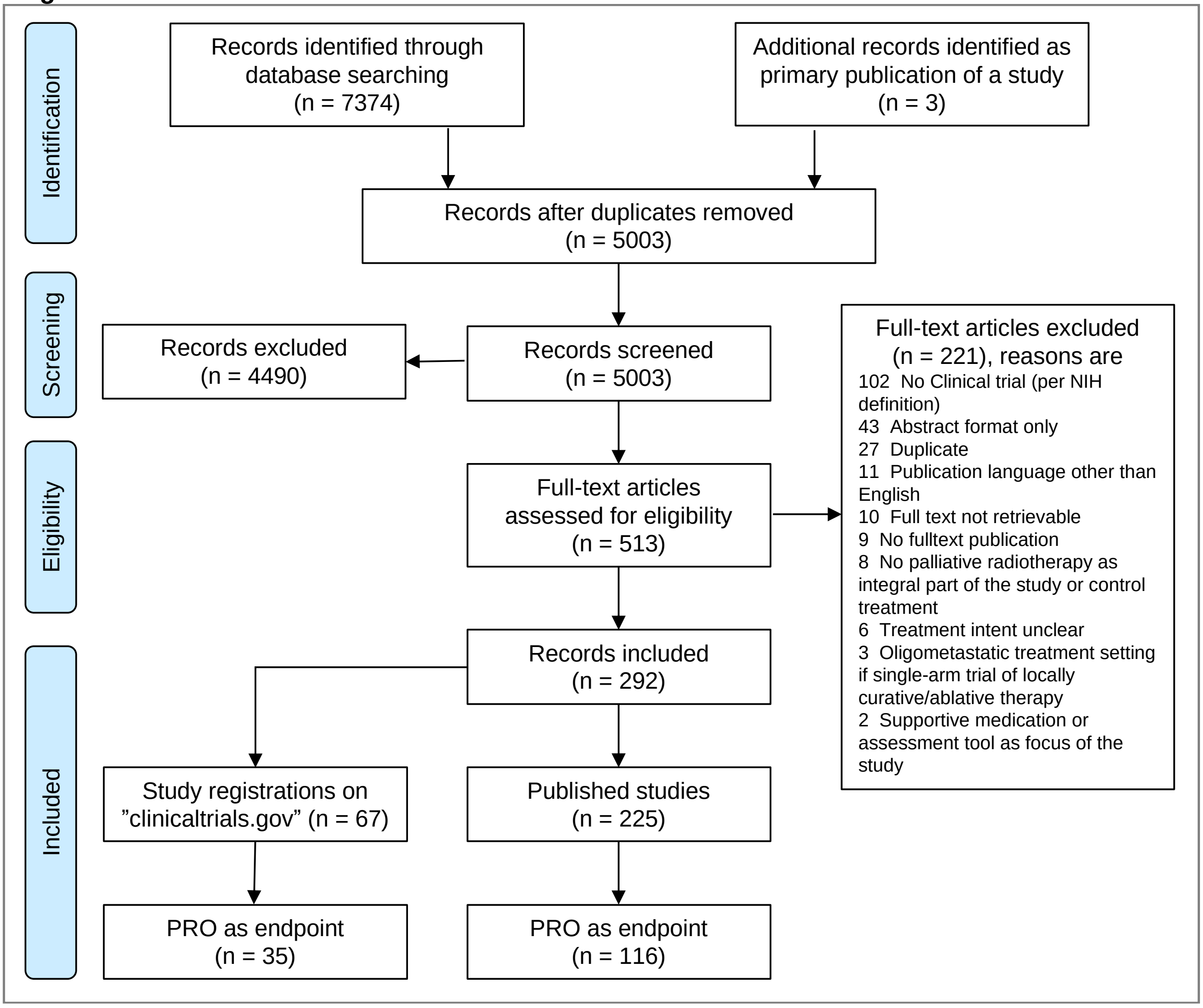

The databases Pubmed/Medline, EMBASE, Cochrane Library, and “ClinicalTrials.gov” were searched. Screening was performed independently by two authors. Included records that belonged to the same trial were merged and counted as one study. Abbreviations: NIH, National Institutes of Health, PRO, patient-reported outcome

© 2022 Fabian A et al. *JAMA Network Open.*

eFigure 2. Patient-Reported Outcomes (PRO) in Published Trials of Palliative Radiotherapy Over Time Separately Analyzed for Randomized and Nonrandomized Trials

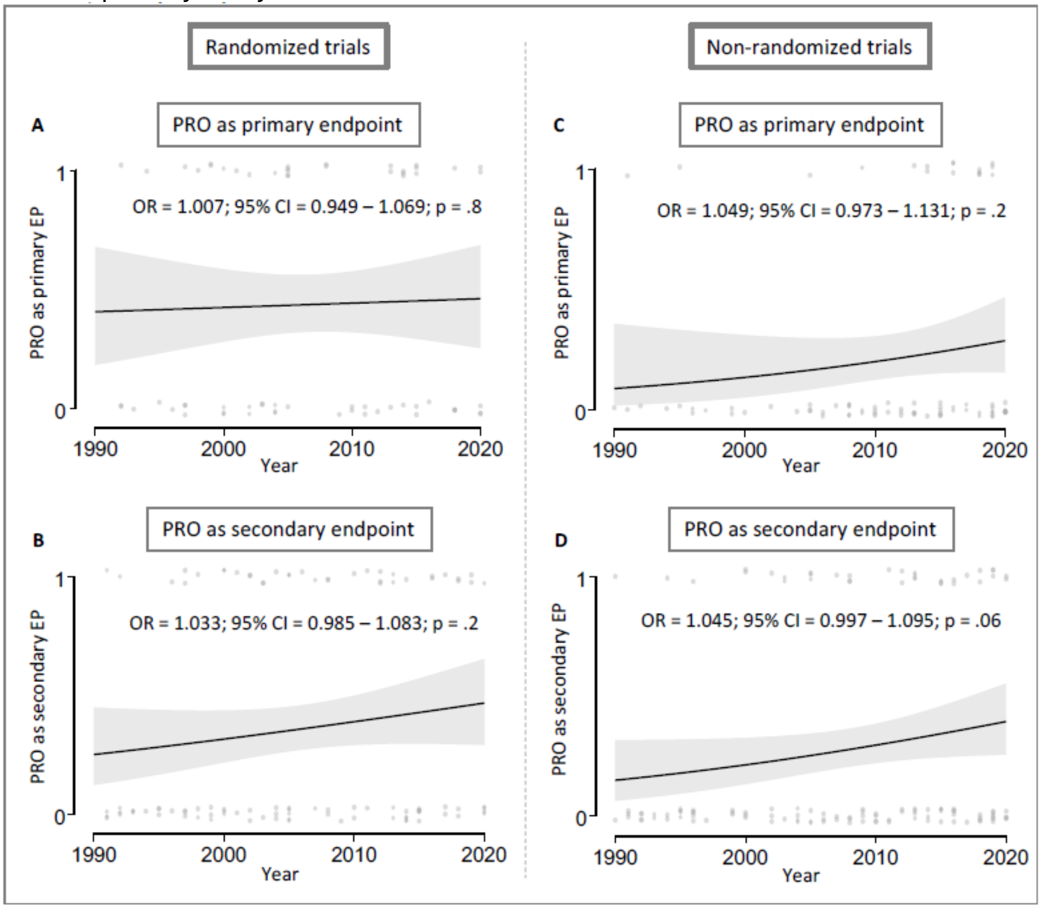

Logistic regression analysis of the use of a patient-reported endpoint (1 = yes, 0 = no) as dependent variable and year of publication as independent variable. Panel A shows the use of PRO as primary endpoint in randomized trials clearly stating their endpoint (n = 64). Panel B shows the use of PRO as secondary endpoint in all randomized trials (n = 100). Panel C shows the use of PRO as primary endpoint in non-randomized trials clearly stating their endpoint (n = 81). Panel D shows the use of PRO as secondary endpoint in all non-randomized trials (n = 125). Grey bullets represent data points. Gray shading around each line represents the respective 95% confidence interval. A *p*-value < .05 was considered statistically significant. Abbreviations: PRO, patient-reported outcome; EP, endpoint

© 2022 Fabian A et al. *JAMA Network Open.*

eFigure 3. Treated Sites in Published Trials of Palliative Radiotherapy Including Patient-Reported Outcomes as Primary (n=45) or Secondary End Points (n=71)

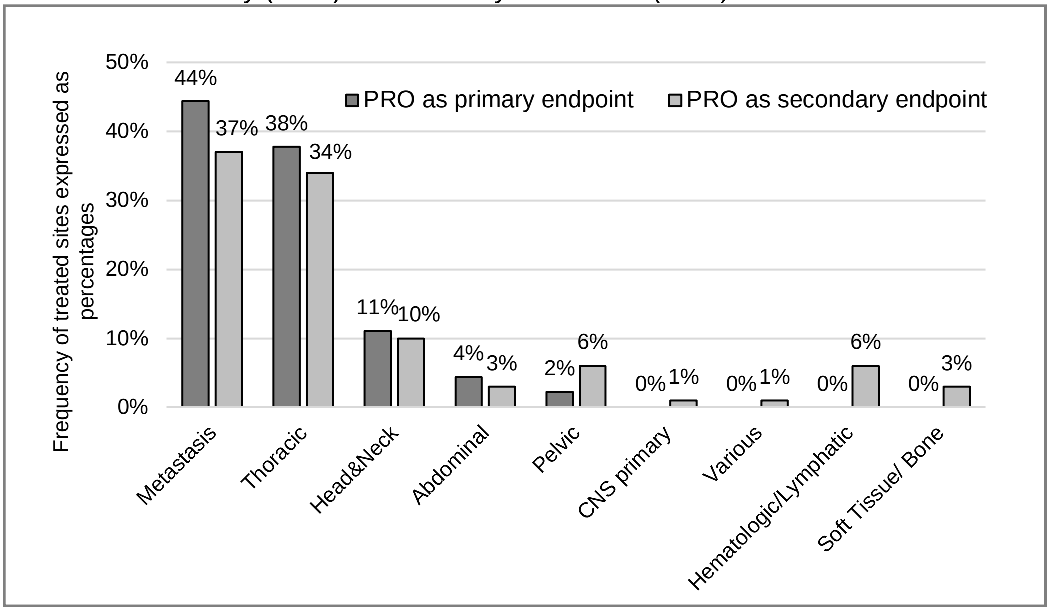

Abbreviation: CNS, central nervous system; PRO, patient-reported outcome

© 2022 Fabian A et al. *JAMA Network Open.*

eTable 1. Summary Parameters of the Multiple Regression Models

Model Summary	Model „extension adherence score“	Model „total CONSROT PRO adherence score”
Number of observations	108	108
F(6,101)	6.660	9.669
Prob > F	<0.001	<0.001
R-squared	0.283	0.365
Adj. R-squared	0.241	0.328
Durbin-Watson-Stat.	1.946	1.966
Variable entry	Simultaneous entry of prespecified variables[a]	
[a]the independent variable “trial phase” was not used due to low case numbers and potential collinearity to the included independent variable “randomization”		

© 2022 Fabian A et al. *JAMA Network Open.*

eTable 2. Factors Associated With the Degree of Patient-Reported Outcome Reporting in Trials of Palliative Radiotherapy

"Extension adherence score" and "total CONSORT-PRO adherence score" are dependent variable and trial characteristics are independent variables in two separate multiple regression models. In this models, the variable "year of publication" was modified to "date of publication > 2013" which represents the publication date of the CONSORT-PRO extension.

	Dependent variable: "extension adherence score"				Dependent variable: "total CONSORT-PRO adherence score"			
Independent variables	B	Lower 95% CI	Upper 95% CI	*p*	B	Lower 95% CI	Upper 95% CI	*p*
(Constant)	6.241	-7.914	20.396	0.384	14.243	1.707	26.779	0.026
Date of publication > 2013 (Yes)	2.022	-5.688	9.733	0.604	3.393	-2.890	10,767	0.255
PRO as primary endpoint (Yes)	9.381	1.819	16.942	**0.016**	12.342	5.646	19.038	**<.001**
Randomization	-0.415	-7.743	8.573	0.920	-1.158	-8.383	6.067	0.751
Multicenter (Yes)	7.514	-0.425	15.453	0.320	7.290	0.259	14.321	**0.042**
Modality of radiotherapy (BT)	17.077	6.091	28.063	**0.003**	14.511	4.782	24.240	**0.004**
Patient number	0.028	0.007	0.050	**0.010**	0.033	0.012	0.050	**0.002**
Abbreviations: B, regression coefficient B; BT, Brachytherapy CI confidence interval								

eTable 3. Summary Parameters of the Multiple Regression Models

Model Summary	Model „extension adherence score"	Model „total CONSROT PRO adherence score"
Number of observations	108	108
F(6,101)	6.713	9.626
Prob > F	<0.001	<0.001
R-squared	0.285	0.364
Adj. R-squared	0.243	0.326
Durbin-Watson-Stat.	2,180	2.273
Variable entry	Simultaneous entry	

© 2022 Fabian A et al. *JAMA Network Open.*

eTable 4. Characteristics of Ongoing Trials Registered on ClinicalTrials.gov With Patient-Reported Outcomes as End Points
Percentages may not add up to 100% (35 registrations) due to rounding error, missing information in registered studies, or multiple values per study.

	Ongoing registered trials with PRO (n=35)
	% (No.)
Study design	
Multicenter	49 (17)
Phase	
I	-
I/II	9 (3)
II	31 (11)
III	20 (7)
Exploratory	9 (3)
Not stated	31 (11)
Randomized	54 (19)
Funding by industry	14 (5)
Location	
N-America	51 (18)
Europe	29 (10)
Asia	14 (5)
Oceania	3 (1)
Multiple	3 (1)
Radiotherapy	
Modality	
EBRT	74 (26)
SRS/SBRT	17 (6)
Other	6 (2)
EBRT with BT	3 (1)
BT	-
Conc. systemic Th.	17 (6)
Immunoth.[a]	9 (3)
Targeted Th.[a]	6 (2)
Chemotherapy	3 (1)

[a]Includes combination with chemotherapy
Abbreviations: BT, brachytherapy; conc., concurrent; EBRT, external beam radiotherapy; PRO, patient-reported outcome; SRS/SBRT, radiosurgery/stereotactic body radiotherapy

© 2022 Fabian A et al. *JAMA Network Open.*

eFigure 4. Treated Sites in Ongoing, Registered Trials of Palliative Radiotherapy Including Patient-Reported Outcomes

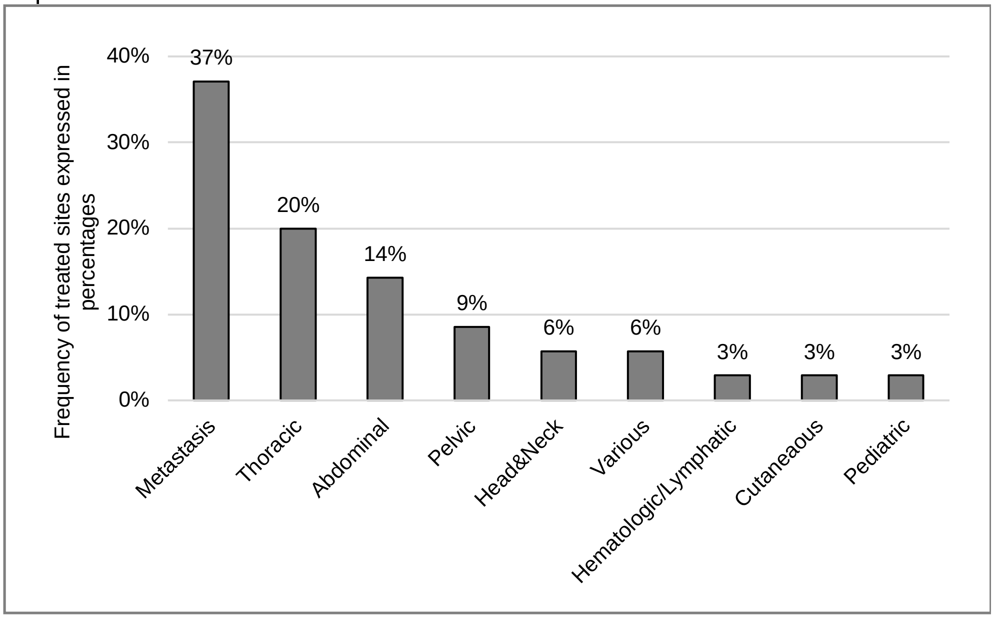

Abbreviation: CNS, central nervous system

Clinical endpoints and patient-reported outcomes in trials of palliative radiotherapy: protocol of a systematic meta-research analysis

A. Fabian[a]#, J. Domschikowski[a], A. Letsch[b], Oliver Weiner[c], C. Schmalz[a], J. Dunst[a], D. Krug[a]

[a]Department of Radiation Oncology, University Hospital Schleswig-Holstein, 24105 Kiel, Germany
[b]Department of Hematology and Oncology, University Hospital Schleswig-Holstein, 24105 Kiel, Germany
[c]University Library Kiel, Christian-Albrechts-University Kiel, Germany

# Correspondence address: Alexander Fabian MD, Department of Radiation Oncology, University Hospital Schleswig-Holstein (UKSH) Campus Kiel, Arnold-Heller-Str. 3, 24105 Kiel, Germany, Tel.: +4943150026501,
Fax: +4943150026564, alexander.fabian(at)uksh.de

Registration

This study will be registered in the "Open Science Framework" (www.osf.io) of the "Center for Open Science".

Author contribution

AF, JD and DK wrote the protocol. AF, JDo, OW, DK, CS, and JDu will be engaged in screening the eligible literature, data extraction and analysis, and drafting a manuscript. Statistical support by S. Freitag-Wolf, statistician, is acknowledged.

Financial Support

Financial support by an unrestricted grant from the non-commercial, academic "Kiel Oncology Network" is acknowledged.

Amendments

Amendments from v1 (27.Feb.2021) to v2 (11.May.2021) are highlighted in yellow. The amendments were introduced at “title/abstract screening” stage.

The amendment from v2 (11.May.2021) to v3 (23.June.2021) is highlighted in green. The amendment was introduced at “full text screening” stage prior to data extraction.

© 2022 Fabian A et al. *JAMA Network Open.*

Index

1. Introduction **4**

1.1. Rationale 4

1.2. Objectives 4

2. Methods **5**

2.1.1 Eligibility criteria 5

2.1.2 Comments on eligibility criteria 6

2.2. Information sources and search strategy 7

2.3. Study records 7

2.4. Data extraction, outcomes and prioritization 7

2.5 Evaluation and statistical analysis plan 8

2.5.1 Primary objective 8

2.5.2 Co-primary objective 9

2.5.3 Secondary objective 10

3. Abbreviations **10**

4. References **10**

5. Appendices **12**

5.1 Definition of relevant terms **12**

5.2 Search strategy 13

5.3 Variables included in data extraction form 1 – "included studies" 13

5.4 Variables included in data extraction form 2 – "CONSORT-PRO" 14

5.5 Variables included in data extraction form 3 – "ongoing studies" 14

© 2022 Fabian A et al. *JAMA Network Open.*

1. Introduction

1.1. Rationale

Palliative radiotherapy is frequently employed in patients suffering from advanced cancers in order to alleviate symptoms and improve health-related quality of life [1] (definition of relevant terms displayed in appendix 1). Multiple prospective studies on palliative radiotherapy have been published in the last decades. Clinical endpoints of prospective clinical trials include "patient-centered clinical endpoints" (overall survival, health-related quality of life, self-reported symptom burden) or "tumor-centered clinical endpoints" (e.g. progression-free survival, local control, radiographic response rate) [2][3]. As most patients face a limited prognosis in terms of survival, the employment of "patient-centered clinical endpoints" in trials of palliative radiotherapy is desirable. Specifically, the impact of palliative radiotherapy on health-related quality of life and symptom burden is paramount as it is for any other palliative care intervention [4]. Health-related quality of life and symptom burden should be measured by patient-reported outcomes (PRO) and in a standardized manner by validated patient-reported outcome measures (PROM) [5].

Palliative radiotherapy is used in various settings including the treatment of patients with symptomatic metastases (e.g. bone or brain metastases) or symptomatic incurable primary cancers (e.g. head and neck cancer or lung cancer). Early guidelines on trials for palliative radiotherapy of bone metastases have fostered the use of "patient-centered clinical endpoints" including patient-reported outcomes in this scenario [6]. In other scenarios, however, "patient-centered clinical endpoints", namely patient-reported outcomes, seem to have been employed less frequently and systematically as demonstrated by our group for studies of palliative radiotherapy for incurable head and neck cancers (Fabian et al. 2020, manuscript submitted).

To date, we lack a systematic overview of the distribution of endpoints employed in clinical trials of palliative radiotherapy across tumor entities. A broader knowledge on the distribution of endpoints chosen in these trials will establish a status quo, help to identify potential gaps concerning "patient-centered clinical endpoints", and foster future patient-centered research.

1.2. Objectives

The primary objective is to determine the distribution of endpoints chosen in prospective trials of palliative radiotherapy. For this purpose, the rate of studies that employed a "patient-centered clinical endpoint" vs. a "tumor-centered clinical endpoint" as primary endpoint will be analyzed.

© 2022 Fabian A et al. *JAMA Network Open.*

Furthermore, the distribution of specific primary and secondary endpoint categories (e.g. overall survival) will be assessed. The distribution of patient-reported outcomes will be highlighted. These rates and distributions will be assessed in the context of prespecified independent variables, for example study design or year of publication (see 2.6 statistical analysis plan).

A co-primary objective is to determine the methodological quality of patient-reported outcome employment in respective studies per CONSORT-PRO reporting guideline [7]. The CONSORT-PRO scores will be assessed in the context of prespecified independent variables, for example study design or year of publication.

The secondary objective is to investigate the distribution of endpoints in ongoing studies per “ClinicalTrials.gov”.

2. Methods

This prospectively planned analysis focusses on patterns in research itself rather than on effect sizes of outcomes in individual studies. Therefore, we perceive this analysis as “meta-research” [8]. Nevertheless, a systematic literature review is the basis of this analysis and the “preferred reporting items for systematic reviews and meta-analysis (PRISMA)” reporting guidelines are followed closely where appropriate [9,10].

2.1.1 Eligibility criteria

The eligibility criteria define studies suitable for final assessment and data extraction according to this study’s objectives.

Inclusion criteria:

- Palliative radiotherapy (including external beam radiotherapy or brachytherapy) as integral part of the study or control treatment
- Cancer patients in a palliative treatment setting
- Clinical trial (per NIH definition [11])
- Trial includes at least one clinical endpoint

© 2022 Fabian A et al. *JAMA Network Open.*

Exclusion criteria:

- Oligometastatic treatment setting if single-arm trial of locally curative/ablative therapy
- Treatment with curative intent
- Supportive medication (e.g. anti-emetics during radiotherapy) or validation of outcome assessment tools as focus of the study
- Abstract format only (e.g. conference talk or poster)
- Publication language other than English

2.1.2 Comments on eligibility criteria

The terms "palliative" and/or "incurable", as required for a study's inclusion, need to be stated and/or defined by the respective study authors.

In case the study population consists of patients with oligometastatic disease (as defined by the respective study authors) the study will only be included for analysis if palliative radiotherapy with non-ablative dosage serves as a control arm. For example, if a study tests stereotactic body radiotherapy compared to palliative radiotherapy in a two-arm trial, this study will be included in case the other eligibility criteria are met. Conversely, if a study consists of a single-arm trial of patients with oligometastatic disease planned to undergo stereotactic body radiotherapy as ablative treatment modality, this study will not be included.

Systemic therapy given concurrently to a palliative radiotherapy regimen, as defined by the authors, is no reason for exclusion.

This paragraph is to further explain the context of eligibility without introducing any change concerning eligibility criteria. The NIH-definition of a "clinical trial" will be strictly applied in the context of palliative radiotherapy. This means that palliative radiotherapy ("palliative" as defined by the respective author) will be considered as *the intervention* in the context of the NIH-definition. The NIH-definition as applied in this analysis is based on four key questions [11]:

1. Does the study involve human participants?
2. Are the participants prospectively assigned to an intervention?
3. Is the study designed to evaluate the effect of *the intervention* on the participants?
4. Is the effect being evaluated a health-related biomedical or behavioral outcome?

For example, if a study mentions palliative radiotherapy as optional in a "standard of care" control arm while the experimental arm investigates a different intervention (e.g. ablative SBRT),

© 2022 Fabian A et al. *JAMA Network Open.*

this study will not be included. Furthermore, observational studies will be largely excluded by this definition as they usually not prospectively assign a patient to an intervention.

2.2. Information sources and search strategy

Sources to screen for eligible studies from 1990 to and including 2020 are PubMed, EMBASE, and Cochrane Central Register of Controlled Trials (CENTRAL). The reason for the limitation in time is to focus on more recent and therefore more relevant changes in the choice of endpoints and employment of PROs by trials. Furthermore, PROs were less established before that period. The search strategy is presented in “appendix 2”. Duplicates will be removed. Furthermore, the trial register “ClinicalTrials.gov” will be screened for ongoing trials (“Recruiting”, “Not yet recruiting”, “active, not recruiting”, “suspended”, “enrolling by invitation”). The search will be conducted by a professional librarian.

2.3. Study records

Search results of the different databases will be merged by a librarian and uploaded to “Covidence”. The software “Covidence” will be employed to manage records. First, at least two authors will independently screen studies based on title and abstract for potential inclusion eligibility. Second, full text will be retrieved. Third, at least two authors will independently judge eligibility based on full text. In case of divergent interpretations concerning eligibility, consensus will be reached by discussion with other co-authors.

2.4. Data extraction, outcomes and prioritization

Data from studies will be extracted based on full text publication. Data collection forms will be pilot-tested for usability and completeness. For this purpose, two authors will independently extract data into prespecified data collection forms of 15 eligible articles. In case of sufficient inter-rater concordance, data extraction of the remaining articles will be continued separately by the authors due to the expected amount of eligible studies and data to extract. ~~At least two authors will independently extract data into prespecified data collection forms.~~ Discrepancies will be resolved by discussion among co-authors.

Endpoints will be assessed per study in case of multiple publications. For example, if there is a publication on efficacy and a separate publication of the same study on quality of life, this will be regarded as one “study”.

© 2022 Fabian A et al. *JAMA Network Open.*

The first form will serve to collect general data as well as endpoints of included studies (variables displayed in appendix 3). In case of multiple co-primary endpoints, all endpoints will be counted as primary endpoints. All endpoints will be defined as secondary endpoints if no clear primary endpoint is defined in the reflective study. The endpoints will be counted per category (patient-centered vs. tumor-centered) and subcategory (e.g. overall survival, health-related quality of life, progression-free survival etc.) as stated in each individual included study (for definitions see appendix 1, for variables see appendix 5).

The second form will be used for studies that employed patient-reported outcomes as an endpoint. The quality level of PRO employment in individual studies will be assessed per CONSORT-PRO reporting guideline as surrogate [7]. Although the CONSORT-PRO guideline is dedicated to randomized trials, its items are equally important for non-randomized trials. This was confirmed by peer review prior to registration of this protocol. There are no dedicated PRO-reporting guidelines for non-randomized studies.

A third form will be used for endpoints in ongoing trials per “ClinicalTrials.gov”.

2.5 Evaluation and statistical analysis plan

Descriptive statistics and testing will be used as appropriate including percentages and means or medians and quartiles.

2.5.1 Primary objective

Independent variables and subgroups for the comparison of trials using “patient-centered clinical endpoints” vs. “tumor-centered clinical endpoints” as primary endpoint include:

- Study design (dedicated phase I vs. II vs III, randomized vs. non-randomized, multi- vs. mono-center)
- Tumor entity (per specific entities, bone metastases vs. other (due to published guidelines for radiotherapy trials of bone metastases)),
- Modality of radiotherapy (external beam radiotherapy vs. brachytherapy)
- Sample size (Number of patients per study)
- Year of publication

© 2022 Fabian A et al. *JAMA Network Open.*

The same independent variables will be considered for display of the distribution of specific endpoints (e.g. overall survival) per primary or secondary endpoint.

2.5.2 Co-primary objective

The quality level of PRO employment in individual studies will be assessed per CONSORT-PRO reporting guideline as surrogate [7]. Although the CONSORT-PRO guideline is dedicated to randomized trials, its items are equally important for non-randomized trials. This was confirmed by peer review prior to registration of this protocol. There are no dedicated PRO-reporting guidelines for non-randomized studies.

Our study adopts without changes a previously described scoring system for the CONSORT-PRO guideline [12]. Accordingly, values for the "CONSORT-PRO score" (max. score = 7 points) and for the "total CONSORT-PRO score" (max score = 14 or 15 points, respectively if PRO secondary or primary endpoint) will be evaluated per study. In case a study employs multiple different PROMs, the overall highest score per CONSORT-PRO item will be used to calculate the summary score. Summary scores will be expressed as percentages of adhered CONSORT-PRO items.

Independent variables for CONSORT-PRO scores for comparisons will include:

- Endpoints status of PRO (primary vs. secondary endpoint)
- Study design (randomized vs. non-randomized, dedicated phase I vs II vs III, multi- vs. mono-center)
- Sample size (Number of patients per study)
- Modality of radiotherapy (external beam radiotherapy vs. brachytherapy)
- Year of publications

Adherence frequencies to each individual item of the CONSORT-PRO guideline will be displayed in percentages per endpoint status of PRO (primary vs. secondary endpoint). Adherence rates will be categorized in poor (≤ 49%), moderate (50-79%), or good (≥ 80%) as described previously [12].

© 2022 Fabian A et al. *JAMA Network Open.*

2.5.3 Secondary objective

The assessment of endpoint distribution in ongoing trials per "clinicalTrials.gov" will be performed on an exploratory basis using descriptive statistics.

3. Abbreviations

PRO, patient-reported outcome; PROM, patient-reported outcome measure

4. References

1. Lutz, S.T.; Jones, J.; Chow, E. Role of Radiation Therapy in Palliative Care of the Patient With Cancer. *J. Clin. Oncol.* **2014**, *32*, 2913–2919, doi:10.1200/JCO.2014.55.1143.
2. Wilson, M.K.; Karakasis, K.; Oza, A.M. Outcomes and Endpoints in Trials of Cancer Treatment: The Past, Present, and Future. *Lancet Oncol.* **2015**, *16*, e32–e42, doi:10.1016/S1470-2045(14)70375-4.
3. Fiteni, F.; Westeel, V.; Pivot, X.; Borg, C.; Vernerey, D.; Bonnetain, F. Endpoints in Cancer Clinical Trials. *J. Visc. Surg.* **2014**, *151*, 17–22, doi:10.1016/j.jviscsurg.2013.10.001.
4. Kelley, A.S.; Morrison, R.S. Palliative Care for the Seriously Ill. *N. Engl. J. Med.* **2015**, *373*, 747–755, doi:10.1056/NEJMra1404684.
5. LeBlanc, T.W.; Abernethy, A.P. Patient-Reported Outcomes in Cancer Care - Hearing the Patient Voice at Greater Volume. *Nat. Rev. Clin. Oncol.* **2017**, *14*, 763–772, doi:10.1038/nrclinonc.2017.153.
6. Chow, E.; Wu, J.S.Y.; Hoskin, P.; Coia, L.R.; Bentzen, S.M.; Blitzer, P.H. International Consensus on Palliative Radiotherapy Endpoints for Future Clinical Trials in Bone Metastases. *Radiother. Oncol. J. Eur. Soc. Ther. Radiol. Oncol.* **2002**, *64*, 275–280, doi:10.1016/s0167-8140(02)00170-6.
7. Calvert, M.; Blazeby, J.; Altman, D.G.; Revicki, D.A.; Moher, D.; Brundage, M.D.; CONSORT PRO Group, for the Reporting of Patient-Reported Outcomes in Randomized Trials: The CONSORT PRO Extension. *JAMA* **2013**, *309*, 814, doi:10.1001/jama.2013.879.
8. Ioannidis, J.P.A.; Fanelli, D.; Dunne, D.D.; Goodman, S.N. Meta-Research: Evaluation and Improvement of Research Methods and Practices. *PLOS Biol.* **2015**, *13*, e1002264, doi:10.1371/journal.pbio.1002264.
9. Moher, D.; Liberati, A.; Tetzlaff, J.; Altman, D.G.; PRISMA Group Preferred Reporting Items for Systematic Reviews and Meta-Analyses: The PRISMA Statement. *BMJ* **2009**, *339*, b2535, doi:10.1136/bmj.b2535.
10. Moher, D.; Shamseer, L.; Clarke, M.; Ghersi, D.; Liberati, A.; Petticrew, M.; Shekelle, P.; Stewart, L.A.; PRISMA-P Group Preferred Reporting Items for Systematic Review and Meta-Analysis Protocols (PRISMA-P) 2015 Statement. *Syst. Rev.* **2015**, *4*, 1, doi:10.1186/2046-4053-4-1.
11. NOT-OD-15-015: Notice of Revised NIH Definition of Clinical Trial Available online: https://grants.nih.gov/grants/guide/notice-files/NOT-OD-15-015.html (accessed on 19 January 2021).
12. Mercieca-Bebber, R.; Rouette, J.; Calvert, M.; King, M.T.; McLeod, L.; Holch, P.; Palmer, M.J.; Brundage, M.; International Society for Quality of Life Research (ISOQOL) Best Practice for PROs—Reporting Taskforce Preliminary Evidence on the Uptake, Use and Benefits

© 2022 Fabian A et al. *JAMA Network Open.*

of the CONSORT-PRO Extension. *Qual. Life Res. Int. J. Qual. Life Asp. Treat. Care Rehabil.* **2017**, *26*, 1427–1437, doi:10.1007/s11136-017-1508-6.
13. Calvert, M.; Kyte, D.; Mercieca-Bebber, R.; Slade, A.; Chan, A.-W.; King, M.T.; and the SPIRIT-PRO Group; Hunn, A.; Bottomley, A.; Regnault, A.; et al. Guidelines for Inclusion of Patient-Reported Outcomes in Clinical Trial Protocols: The SPIRIT-PRO Extension. *JAMA* **2018**, *319*, 483, doi:10.1001/jama.2017.21903.
14. Biomarkers Definitions Working Group Biomarkers and Surrogate Endpoints: Preferred Definitions and Conceptual Framework. *Clin. Pharmacol. Ther.* **2001**, *69*, 89–95, doi:https://doi.org/10.1067/mcp.2001.113989.

© 2022 Fabian A et al. *JAMA Network Open.*

5. Appendices

5.1 Definition of relevant terms

Term	Definition[13]
Clinical endpoint	A characteristic or variable that reflects how a patient feels, functions, or survives. [14]
Clinical trial	A research study in which one or more human subjects are prospectively assigned to one or more interventions (which may include placebo or other control) to evaluate the effects of those interventions on health-related biomedical or behavioral outcomes. [11]
Health-related quality of life	A multidimensional concept that usually includes self-report of the way in which physical, emotional, social, or other domains of well-being are affected by a disease or its treatment. [13]
Patient-reported outcome (PRO)	An outcome reported directly by patients themselves and not interpreted by an observer; PROs may include patient assessments of health status, quality of life, or symptoms. [13]
Patient-centered endpoint	Clinically meaningful endpoint. Including: overall survival, patient-reported outcome for health-related quality of life or symptom burden. [2,3]
Patient-reported outcome measure (PROM)	Instrument to measure PROs (e.g. multi-item questionnaires)
Primary outcome/end point	The most important outcome in a trial, providing the most clinically relevant evidence directly related to the primary objective of the trial. [13]
Secondary outcome / end point	Outcomes prespecified in the protocol to assess additional effects of the intervention. [13] All endpoints if no primary endpoint is defined (see 2.4)
Tumor-centered endpoints	(Surrogate) Endpoint that result (in part) of radiographical, biochemical, or clinical tumor activity and aim to substitute or complement patient-centered endpoints. [2,3] Including but not limited to: progression-free

© 2022 Fabian A et al. *JAMA Network Open.*

	survival, disease-free survival, recurrence-free survival, event-free survival, failure-free survival, local control/recurrence, radiographic/clinical (other than patient-reported)/biochemical tumor response rate, remission rate, time to new treatment.

PICOS	Layer	Search string (exemplified for PubMed/Medline)
Population	#1	Palliative care[MeSH] OR Terminally Ill[Mesh] OR palliat*[title/abstract] OR incurable[title/abstract]
Intervention	#2	radiotherapy[MeSH] OR radiotherapy[title/abstract] OR "radiation therapy" [title/abstract] OR radiotherap*[title/abstract] OR radiation[title/abstract] OR irradiat*[title/abstract] OR brachytherapy[title/abstract] OR chemoradiotherap*[title/abstract] OR radiochemotherap*[title/abstract] OR chemoradiat*[title/abstract]
Comparator	not applicable	
Outcome	not specified	
Study type	#3	prospective study [MeSH] OR clinical study[publication type] OR clinical trial[publication type] OR prospective*[tw] OR trial[title/abstract] OR phase[title/abstract]
	#4	#1 AND #2 AND #3

5.2 Search strategy

5.3 Variables included in data extraction form 1 – "included studies"

Category	Variables
General	first author, year, location
Study design	phase, arms, randomization, specific indication, specific radiotherapy regimen, number of centers, concurrent antineoplastic systemic per protocol, control arm of oligo-metastatic cancer study, patient number, age, performance status, study sponsor ("industry" if at least one sponsor from industry; otherwise "academic") cancer entity
Endpoint category	primary, secondary
Endpoint type	overall survival, health related quality of life per patient reported outcomes, symptom control per patient reported outcomes (including symptomatic response

© 2022 Fabian A et al. *JAMA Network Open.*

	rate), progression free survival, disease-free survival, recurrence-free survival, local control, toxicity, objective response rate, radiographic response rate, biochemical response rate, toxicity, change in performance status, feasibility, other

5.4 Variables included in data extraction form 2 – "CONSORT-PRO"

Category	Variables
General	first author, year
Study design	randomization, CONSORT-PRO cited, name of PROM, number of PROMs
CONSORT-PRO checklist	scoring as described in [12]

5.5 Variables included in data extraction form 3 – "ongoing studies"

Category	Variables
General	study identifier, location, tumor entity, start date, status, planned patient number, actual patient number, study design
Endpoint category	primary, secondary
Endpoint type	overall survival, health related quality of life per patient reported outcomes, symptom control per patient reported outcomes (including symptomatic response rate), progression free survival, disease-free survival, recurrence-free survival, local control, toxicity, objective response rate, radiographic response rate, biochemical response rate, toxicity, change in performance status, feasibility, other

© 2022 Fabian A et al. *JAMA Network Open.*

eAppendix 2. References of Included Published Trials of Palliative Radiotherapy and Trial Registrations

The reference list of published trials includes trials with multiple publications which were merged to one record. Therefore, it exceeds the number indicated in the PRISMA flow chart.

1. Abratt RP, Shepherd LJ, Memeena Salton DG. Palliative radiation for stage 3 non-small cell lung cancer - A prospective study of two moderately high dose regimens. *Lung Cancer*. 1995;13(2):137-143. doi:10.1016/0169-5002(95)00487-4

2. Aggarwal R, Patel FD, Kapoor R, Kang M, Kumar P, Chander Sharma S. Evaluation of high-dose-rate intraluminal brachytherapy by percutaneous transhepatic biliary drainage in the palliative management of malignant biliary obstruction-A pilot study. *Brachytherapy*. 2013;12(2):162-170. doi:10.1016/j.brachy.2012.06.002

3. Akl FM, Elsayed-Abd-Alkhalek S, Salah T. Palliative concurrent chemoradiotherapy in locally advanced and metastatic esophageal cancer patients with dysphagia. *Ann Palliat Med*. 2013;2(3):118-123. doi:10.3978/j.issn.2224-5820.2013.05.01

4. Al-Mamgani A, Kessels R, Verhoef CG, et al. Randomized controlled trial to identify the optimal radiotherapy scheme for palliative treatment of incurable head and neck squamous cell carcinoma. *Radiother Oncol*. 2020;149:181-188. doi:10.1016/j.radonc.2020.05.020

5. Ali AM, El-Sayed MI. Metronomic chemotherapy and radiotherapy as salvage treatment in refractory or relapsed pediatric solid tumours. *Curr Oncol*. 2016;23(3):e253-9. doi:10.3747/co.23.2873

6. Amdal CD, Jacobsen AB, Sandstad B, Warloe T, Bjordal K. Palliative brachytherapy with or without primary stent placement in patients with oesophageal cancer, a randomised phase III trial. *Radiother Oncol*. 2013;107(3):428-433. doi:10.1016/j.radonc.2013.04.008

7. Amouzegar-Hashemi F, Behrouzi H, Kazemian A, Zarpak B, Haddad P. Single versus multiple fractions of palliative radiotherapy for bone metastases: A randomized clinical trial in Iranian patients. *Curr Oncol*. 2008;15(3):36-39. doi:10.3747/co.v15i3.203

8. André T, Balosso J, Louvet C, et al. Combined radiotherapy and chemotherapy (cisplatin and 5-fluorouracil) as palliative treatment for localized unresectable or adjuvant treatment for resected pancreatic adenocarcinoma: Results of a feasibility study. *Int J Radiat Oncol Biol Phys*. 2000;46(4):903-911. doi:10.1016/S0360-3016(99)00478-2

9. Anter AH. Single fraction versus multiple fraction radiotherapy for treatment of painful bone metastases: A prospective study; Mansoura experience. *Forum Clin Oncol*. 2015;6(2):8-13. doi:10.1515/fco-2015-0007

10. Asghar AH, Mahmood H, Faheem M, Rizvi S, Sajid A, Irfan J. Therapeutic effects of 20 gray radiotherapy over a week in the management of brain metastases. *JPMA J Pak Med Assoc*. 2009;59(5):278-281.

11. Atahan L, Yildiz F, Cengiz M, et al. Zoledronic acid concurrent with either high-or reduced-dose palliative radiotherapy in the management of the breast cancer patients with bone metastases: A phase IV randomized clinical study. *Support Care Cancer*. 2010;18(6):691-698. doi:10.1007/s00520-009-0663-x

12. Azria D, Ychou M, Jacot W, et al. Treatment of unresectable, locally advanced pancreatic adenocarcinoma with combined radiochemotherapy with 5-fluorouracil and cisplatin. *Pancreas*. 2002;25(4):360-365. doi:10.1097/00006676-200211000-00007

13. Baczyk M, Milecki P, Pisarek M, Gut P, Antczak A, Hrab M. A prospective randomized trial: a comparison of the analgesic effect and toxicity of 153Sm radioisotope treatment in monotherapy and combined therapy including local external beam radiotherapy (EBRT) among metastatic castrate resistance prostate cancer (mCRPC) patients with painful bone metastases. *Neoplasma*. 2013;60(3):328-333. doi:10.4149/neo_2013_044

14. Badzio A, Senkus-Konefka E, Jereczek-Fossa BA, et al. 20 Gy in five fractions versus 8 Gy in one fraction in palliative radiotherapy of bone metastases. A multicenter randomized study. *Nowotwory*. 2003;53(3):261-264.

15. Balducci M, Diletto B, Chiesa S, et al. Low-dose fractionated radiotherapy and concomitant chemotherapy for recurrent or progressive glioblastoma: final report of a pilot study. *Strahlenther Onkol*. 2014;190(4):370-376. doi:10.1007/s00066-013-0506-z

16. Ball D, Smith J, Bishop J, et al. A phase III study of radiotherapy with and without continuous-infusion fluorouracil as palliation for non-small-cell lung cancer. *Br J Cancer*. 1997;75(5):690-697. doi:10.1038/bjc.1997.123

17. Barroso-Sousa R, Krop IE, Trippa L, et al. A Phase II Study of Pembrolizumab in Combination With Palliative Radiotherapy for Hormone Receptor-positive Metastatic Breast Cancer. *Clin Breast Cancer*. 2020;20(3):238-245. doi:10.1016/j.clbc.2020.01.012

18. Bauman GS, Gaspar LE, Fisher BJ, Halperin EC, Macdonald DR, Cairncross JG. A prospective study of short-course radiotherapy in poor prognosis glioblastoma multiforme. *Int J Radiat Oncol Biol Phys*. 1994;29(4):835-839.

19. Bebb G, Smith C, Rorke S, et al. Phase i clinical trial of the anti-EGFR monoclonal antibody nimotuzumab with concurrent external thoracic radiotherapy in Canadian patients diagnosed with stage IIb, III or IV non-small cell lung cancer unsuitable for radical therapy. *Cancer Chemother Pharmacol*. 2011;67(4):837-845. doi:10.1007/s00280-010-1379-9

20. Bergquist H, Johnsson E, Nyman J, et al. Combined stent insertion and single high-dose brachytherapy in patients with advanced esophageal cancer - results of a prospective safety study. *Dis Esophagus*. 2012;25(5):410-415. doi:10.1111/j.1442-2050.2011.01248.x

21. Bergquist H, Wenger U, Johnsson E, et al. Stent insertion or endoluminal brachytherapy as palliation of patients with advanced cancer of the esophagus and gastroesophageal junction. Results of a randomized, controlled clinical trial. *Dis Esophagus*. 2005;18(3):131-139. doi:10.1111/j.1442-2050.2005.00467.x

22. Berwouts D, De Wolf K, Lambert B, et al. Biological 18[F]-FDG-PET image-guided dose painting by numbers for painful uncomplicated bone metastases: A 3-arm randomized phase II trial. *Radiother Oncol*. 2015;115(2):272-278. doi:10.1016/j.radonc.2015.04.022

23. Bezjak A, Dixon P, Brundage M, et al. Randomized phase III trial of single versus fractionated thoracic radiation in the palliation of patients with lung cancer (NCIC CTG SC.15). *Int J Radiat Oncol Biol Phys*. 2002;54(3):719-728. doi:10.1016/S0360-3016(02)02989-9

24. Bissett D, Macbeth FR, Cram I. The role of palliative radiotherapy in malignant mesothelioma. *Clin Oncol*. 1991;3(6):315-317.

25. Biswal BM, Ruzman N, Ahmad NM, Zakaria A. Split-Course Radiotherapy in Stage IV Head & Neck Cancer. *Malays J Med Sci*. 2000;7(1):54-59.

26. Bleehen NM, Girling DJ, Machin D, Stephens RJ. A Medical Research Council (MRC) randomised trial of palliative radiotherapy with two fractions or a single fraction in patients with inoperable non-small-cell lung cancer (NSCLC) and poor performance status. *Br J Cancer*. 1992;65(6):934-941. doi:10.1038/bjc.1992.196

27. Bleehen NM, Girling DJ, Fayers PM, Aber VR, Stephens RJ. Inoperable non-small-cell lung cancer (NSCLC): A Medical Research Council randomised trial of palliative radiotherapy with two fractions or ten fractions. *Br J Cancer*. 1991;63(2):265-270.

28. Bludau F, Welzel G, Reis T, et al. Phase I/II trial of combined kyphoplasty and intraoperative radiotherapy in spinal metastases. *Spine J*. 2018;18(5):776-781. doi:10.1016/j.spinee.2017.09.011

29. Borg D, Sundberg J, Brun E, et al. Palliative short-course hypofractionated radiotherapy followed by chemotherapy in esophageal adenocarcinoma: the phase II PALAESTRA trial. *Acta Oncol*. 2020;59(2):212-218. doi:10.1080/0284186X.2019.1670861

30. Brada M, Thomas G, Elyan S, et al. Improving the acceptability of high-dose radiotherapy by reducing the duration of treatment: Accelerated radiotherapy in high-grade glioma. *Br J Cancer*. 1995;71(6):1330-1334.

31. Burmeister BH, Denham JW, O'Brien M, et al. Combined modality therapy for esophageal carcinoma: Preliminary results from a large Australasian multicenter study. *Int J Radiat Oncol Biol Phys*. 1995;32(4):997-1006. doi:10.1016/0360-3016(94)00449-U

32. Burmeister BH, Walpole ET, Burmeister EA, et al. Feasibility of chemoradiation therapy with protracted infusion of 5-fluorouracil for esophageal cancer patients not suitable for cisplatin. *Int J Clin Oncol*. 2005;10(4):256-261. doi:10.1007/s10147-005-0506-9

33. Bydder S, Spry NA, Christie DRH, et al. A prospective trial of short-fractionation radiotherapy for the palliation of liver metastases. *Australas Radiol*. 2003;47(3):284-288. doi:10.1046/j.1440-1673.2003.01177.x

34. Čanak V, Zarić B, Milovančev A, et al. Combination of interventional pulmonology techniques (Nd:YAG laser resection and brachytherapy) with external beam radiotherapy in the treatment of lung cancer patients with Karnofsky Index ≤ 50. *J BUON*. 2006;11(4):447-456.

35. Capuccini J, Macchia G, Farina E, et al. Short-course regimen of palliative radiotherapy in complicated bone metastases: a phase i–ii study (SHARON Project). *Clin Exp Metastasis*. 2018;35(7):605-611. doi:10.1007/s10585-018-9931-9

36. Caravatta L, Deodato F, Ferro M, et al. A phase I study of short-course accelerated whole brain radiation therapy for multiple brain metastases. *Int J Radiat Oncol Biol Phys*. 2012;84(4):e463-e468. doi:10.1016/j.ijrobp.2012.06.023

37. Caravatta L, Deodato F, Ferro M, et al. Results of a Phase II Study of Short-Course Accelerated Radiation Therapy (SHARON) for Multiple Brain Metastases. *Am J Clin Oncol*. 2015;38(4):395-400. doi:10.1097/COC.0b013e3182a0e826

38. Caravatta L, Padula GDA, MacChia G, et al. Short-course accelerated radiotherapy in palliative treatment of advanced pelvic malignancies: A phase i study. *Int J Radiat Oncol Biol Phys*. 2012;83(5):e627-e631. doi:10.1016/j.ijrobp.2011.10.081

39. Carrascosa LA, Yashar CM, Paris KJ, Larocca RV, Faught SR, Spanos WJ. Palliation of pelvic and head and neck cancer with paclitaxel and a novel radiotherapy regimen. *J Palliat Med*. 2007;10(4):877-881. doi:10.1089/jpm.2006.0192

40. Chanana AD, Capala J, Chadha M, et al. Boron neutron capture therapy for glioblastoma multiforme: Interim results from the phase I/II dose-escalation studies. *Neurosurgery*. 1999;44(6):1182-1193. doi:10.1097/00006123-199906000-00013

41. Chella A, Ambrogi MC, Ribechini A, et al. Combined Nd-YAG laser/HDR brachytherapy versus Nd-YAG laser only in malignant central airway involvement: A prospective randomized study. *Lung Cancer*. 2000;27(3):169-175. doi:10.1016/S0169-5002(99)00102-6

42. Chen Y, Wang X, Yan Z, et al. The use of ^{125}I seed strands for intraluminal brachytherapy of malignant obstructive jaundice. *Cancer Biother Radiopharm*. 2012;27(5):317-323. doi:10.1089/cbr.2011.0999

43. Chen Y, Wang XL, Yan ZP, et al. HDR-192Ir intraluminal brachytherapy in treatment of malignant obstructive jaundice. *World J Gastroenterol*. 2004;10(23):3506-3510. doi:10.3748/wjg.v10.i23.3506

© 2022 Fabian A et al. *JAMA Network Open.*

44. Cho SH, Shim HJ, Lee SR, et al. Concurrent chemoradiotherapy with S-1 and cisplatin in advanced esophageal cancer. *Dis Esophagus*. 2008;21(8):697-703. doi:10.1111/j.1442-2050.2008.00837.x

45. Choi B, Robins HI, Schiller J, Mehta M. A phase I study of gemcitabine plus palliative radiation therapy for advanced lung cancer. *Cancer Chemother Pharmacol*. 2008;63(1):175-179. doi:10.1007/s00280-008-0708-8

46. Choi HJ, Sohn JH, Lee CG, et al. A phase I study of nimotuzumab in combination with radiotherapy in stages IIB-IV non-small cell lung cancer unsuitable for radical therapy: Korean results. *Lung Cancer*. 2011;71(1):55-59. doi:10.1016/j.lungcan.2010.04.010

47. Choudhary A, Gupta A. Conventional fractionation versus quad shot in advanced head-and-neck cancers: a randomized controlled trial. *Indian J Palliat Care*. 2019;25(4):527-534.

48. Chow E, van der Linden YM, Roos D, et al. Single versus multiple fractions of repeat radiation for painful bone metastases: A randomised, controlled, non-inferiority trial. *Lancet Oncol*. 2014;15(2):164-171. doi:10.1016/S1470-2045(13)70556-4

49. Chow R, Ding K, Ganesh V, et al. Gender and age make no difference in the re-irradiation of painful bone metastases: A secondary analysis of the NCIC CTG SC.20 randomized trial. *Radiother Oncol*. 2018;126(3):541-546. doi:10.1016/j.radonc.2017.10.006

50. Coia LR, Engstrom PF, Paul AR, Stafford PM, Hanks GE. Long-term results of infusional 5-FU, mitomycin-C, and radiation as primary management of esophageal carcinoma. *Int J Radiat Oncol Biol Phys*. 1991;20(1):29-36.

51. Corry J, Peters LJ, D'Costa I, et al. The "QUAD SHOT" - A phase II study of palliative radiotherapy for incurable head and neck cancer. *Radiother Oncol*. 2005;77(2):137-142. doi:10.1016/j.radonc.2005.10.008

52. Cross CK, Berman S, Buswell L, Johnson B, Baldini EH. Prospective study of palliative hypofractionated radiotherapy (8.5 Gy x 2) for patients with symptomatic non-small-cell lung cancer. *Int J Radiat Oncol Biol Phys*. 2004;58(4):1098-1105. doi:10.1016/j.ijrobp.2003.08.005

53. de Wit R, Smit WG, Veenhof KH, Bakker PJ, Oldenburger F, González DG. Palliative radiation therapy for AIDS-associated Kaposi's sarcoma by using a single fraction of 800 cGy. *Radiother Oncol*. 1990;19(2):131-136. doi:10.1016/0167-8140(90)90126-h

54. Denham JW, Hamilton CS, Simpson SA, et al. Acute reaction parameters for human oropharyngeal mucosa. *Radiother Oncol*. 1995;35(2):129-137. doi:10.1016/0167-8140(95)01545-r

55. Denham JW, Burmeister BH, Lamb DS, et al. Factors influencing outcome following radio-chemotherapy for oesophageal cancer. *Radiother Oncol*. 1996;40(1):31-43. doi:10.1016/0167-8140(96)01762-8

56. Deressa BT, Tigeneh W, Bogale N, Buwenge M, Morganti AG, Farina E. Short-Course 2-Dimensional Radiation Therapy in the Palliative Treatment of Esophageal Cancer in a Developing Country: A Phase II Study (Sharon Project). *Int J Radiat Oncol Biol Phys*. 2019;((Deressa B.T.; Tigeneh W.; Bogale N.) Radiotherapy Department, Tikur Anbessa Specialized Hospital, Addis Ababa, Ethiopia(Deressa B.T.) Department of Clinical Oncology, College of Medicine and Health Sciences, University of Gondar, Gondar, Ethiopia(Buwenge). doi:10.1016/j.ijrobp.2019.10.004

57. Dhir V, Swaroop VS, Mohandas KM, et al. Combination chemotherapy and radiation for palliation of hepatocellular carcinoma. *Am J Clin Oncol*. 1992;15(4):304-307. doi:10.1097/00000421-199208000-00005

© 2022 Fabian A et al. *JAMA Network Open.*

58. Di Lorenzo G, Autorino R, Ciardiello F, et al. External beam radiotherapy in bone metastatic prostate cancer: impact on patients' pain relief and quality of life. *Oncol Rep*. 2003;10(2):399-404.

59. Dritschilo A, Huang CH, Rudin CM, et al. Phase I study of liposome-encapsulated c-raf antisense oligodeoxyribonucleotide infusion in combination with radiation therapy in patients with advanced malignancies. *Clin Cancer Res*. 2006;12(4):1251-1259. doi:10.1158/1078-0432.CCR-05-1260

60. Duchesne GM, Bolger JJ, Griffiths GO, et al. A randomized trial of hypofractionated schedules of palliative radiotherapy in the management of bladder carcinoma: Results of medical research council trial BA09. *Int J Radiat Oncol Biol Phys*. 2000;47(2):379-388. doi:10.1016/S0360-3016(00)00430-2

61. Duvall GA, Tarabar D, Seidel RH, Elstad NL, Fowers KD. Phase 2: A dose-escalation study of OncoGel (ReGel/paclitaxel), a controlled-release formulation of paclitaxel, as adjunctive local therapy to external-beam radiation in patients with inoperable esophageal cancer. *Anticancer Drugs*. 2009;20(2):89-95. doi:10.1097/CAD.0b013e3283222c12

62. Eldeeb NA, Bela AM, Eganady AA, Radwan AS. Comparative study of two radiotherapy regimens for palliation of symptomatic advanced non-small cell lung cancer. *Egypt J Chest Dis Tuberc*. 2014;63(2):423-434. doi:10.1016/j.ejcdt.2013.12.006

63. Erridge SC, Gaze MN, Price A, et al. Symptom control and quality of life in people with lung cancer: A randomised trial of two palliative radiotherapy fractionation schedules. *Clin Oncol*. 2005;17(1):61-67. doi:10.1016/j.clon.2004.09.008

64. Falk SJ, Girling DJ, White RJ, et al. Immediate versus delayed palliative thoracic radiotherapy in patients with unresectable locally advanced non-small cell lung cancer and minimal thoracic symptoms: Randomised controlled trial. *Br Med J*. 2002;325(7362):465-468.

65. Farina E, Capuccini J, Macchia G, et al. Phase I-II study of short-course accelerated radiotherapy (SHARON) for palliation in head and neck cancer. *Anticancer Res*. 2018;38(4):2409-2414. doi:10.21873/anticanres.12491

66. Farina E, Macchia G, Buwenge M, et al. Radiotherapy in palliation of thoracic tumors: a phase I–II study (SHARON project). *Clin Exp Metastasis*. 2018;35(8):739-746. doi:10.1007/s10585-018-9942-6

67. Farina E, Macchia G, Siepe G, et al. Palliative short-course radiotherapy in advanced pelvic cancer: A phase II study (SHARON project). *Anticancer Res*. 2019;39(8):4237-4242. doi:10.21873/anticanres.13585

68. Faul C, Gerszten K, Edwards R, et al. A phase I/II study of hypofractionated whole abdominal radiation therapy in patients with chemoresistant ovarian carcinoma: Karnofsky score determines treatment outcome. *Int J Radiat Oncol Biol Phys*. 2000;47(3):749-754. doi:10.1016/S0360-3016(00)00446-6

69. Ferro M, Macchia G, Re A, et al. Advanced head and neck cancer in older adults: Results of a short course accelerated radiotherapy trial. *J Geriatr Oncol*. 2020;((Ferro M., milena.ferro@gemellimolise.it; Macchia G.; Re A.; Ferro M.; Boccardi M.; Picardi V.; Arena E.; Deodato F.) Radiation Oncology Unit, Gemelli Molise Hospital, Catholic University of Sacred Heart, Largo A. Gemelli 1, Campobasso, Italy(Buwenge M.;). doi:10.1016/j.jgo.2020.10.006

70. Foro Arnalot P, Fontanals AV, Galcerán JC, et al. Randomized clinical trial with two palliative radiotherapy regimens in painful bone metastases: 30 Gy in 10 fractions compared with 8 Gy in single fraction. *Radiother Oncol*. 2008;89(2):150-155. doi:10.1016/j.radonc.2008.05.018

71. Fortin B, Khaouam N, Filion E, Nguyen-Tan PF, Bujold A, Lambert L. Palliative radiation therapy for advanced head and neck carcinomas: A phase 2 study. *Int J Radiat Oncol Biol Phys*. 2016;95(2):647-653. doi:10.1016/j.ijrobp.2016.01.039

© 2022 Fabian A et al. *JAMA Network Open.*

72. Franco P, Migliaccio F, Angelini V, et al. Palliative radiotherapy for painful bone metastases from solid tumors delivered with static ports of tomotherapy (TomoDirect): Feasibility and clinical results. *Cancer Invest*. 2014;32(9):458-463. doi:10.3109/07357907.2014.958495

73. Furlan C, Canzonieri V, Spina M, et al. Low-dose radiotherapy in diffuse large B-cell lymphoma. *Hematol Oncol*. 2017;35(4):472-479. doi:10.1002/hon.2368

74. Gaze MN, Kelly CG, Kerr GR, et al. Pain relief and quality of life following radiotherapy for bone metastases: A randomised trial of two fractionation schedules. *Radiother Oncol*. 1997;45(2):109-116. doi:10.1016/S0167-8140(97)00101-1

75. Ghoshal S, Chakraborty S, Moudgil N, Kaur M, Patel FD. Quad shot: a short but effective schedule for palliative radiation for head and neck carcinoma. *Indian J Palliat Care*. 2009;15(2):137-140. doi:10.4103/0973-1075.58460

76. Guden M, Kurt E, Ulutin C. Six gray single dose radiotherapy in the treatment of metastatic bone pain. *Tohoku J Exp Med*. 2002;197(2):111-114. doi:10.1620/tjem.197.111

77. Guerrieri M, Wong K, Ryan G, Millward M, Quong G, Ball DL. A randomised phase III study of palliative radiation with concomitant carboplatin for brain metastases from non-small cell carcinoma of the lung. *Lung Cancer*. 2004;46(1):107-111. doi:10.1016/j.lungcan.2004.02.019

78. Gutiérrez Bayard L, Salas Buzón Mdel C, Angulo Paín E, de Ingunza Barón L. Radiation therapy for the management of painful bone metastases: Results from a randomized trial. *Rep Pract Oncol Radiother*. 2014;19(6):405-411. doi:10.1016/j.rpor.2014.04.009

79. Haas RLM, Poortmans Ph, De Jong D, et al. Effective palliation by low dose local radiotherapy for recurrent and/or chemotherapy refractory non-follicular lymphoma patients. *Eur J Cancer*. 2005;41(12):1724-1730. doi:10.1016/j.ejca.2005.04.033

80. Haie-Meder C, Pellae-Cosset B, Laplanche A, et al. Results of a randomized clinical trial comparing two radiation schedules in the palliative treatment of brain metastases. *Radiother Oncol*. 1993;26(2):111-116.

81. Hamouda WE, Roshdy W, Teema M. Single versus conventional fractionated radiotherapy in the palliation of painful bone metastases. *Gulf J Oncolog*. 2007;1(1):35-41.

82. Haraf DJ, Vokes EE, Panje WR, Weichselbaum RR. Survival and analysis of failure following hydroxyurea, 5-fluorouracil and concomitant radiation therapy in poor prognosis head and neck cancer. *Am J Clin Oncol*. 1991;14(5):419-426. doi:10.1097/00000421-199110000-00012

83. Harrington KJ, Karapanagiotou EM, Roulstone V, et al. Two-stage phase I dose-escalation study of intratumoral reovirus type 3 dearing and palliative radiotherapy in patients with advanced cancers. *Clin Cancer Res*. 2010;16(11):3067-3077. doi:10.1158/1078-0432.CCR-10-0054

84. Harstell WF, Scott CB, Bruner DW, et al. Randomized trial of short- versus long-course radiotherapy for palliation of painful bone metastases. *J Natl Cancer Inst*. 2005;97(11):798-804. doi:10.1093/jnci/dji139

85. Hayter CRR, Huff-Winters C, Paszat L, Youssef YM, Shelley WE, Schulze K. A prospective trial of short-course radiotherapy plus chemotherapy for palliation of dysphagia from advanced esophageal cancer. *Radiother Oncol*. 2000;56(3):329-333. doi:10.1016/S0167-8140(00)00225-5

86. He J, Shi S, Ye L, et al. A randomized trial of conventional fraction versus hypofraction radiotherapyfor bone metastases from hepatocellular carcinoma. *J Cancer*. 2019;10(17):4031-4037. doi:10.7150/jca.28674

87. Hernandez P, Gursahaney A, Roman T, et al. High dose rate brachytherapy for the local control of endobronchial carcinoma following external irradiation. *Thorax*. 1996;51(4):354-358. doi:10.1136/thx.51.4.354

© 2022 Fabian A et al. *JAMA Network Open.*

88. Hirdes MMC, Van Hooft JE, Wijrdeman HK, et al. Combination of biodegradable stent placement and single-dose brachytherapy is associated with an unacceptably high complication rate in the treatment of dysphagia from esophageal cancer. *Gastrointest Endosc*. 2012;76(2):267-274. doi:10.1016/j.gie.2012.04.442

89. Hoegler DB, Davey P. A prospective study of short course radiotherapy in elderly patients with malignant glioma. *J Neurooncol*. 1997;33(3):201-204. doi:10.1023/A:1005750111883

90. Homs MYV, Essink-Bot ML, Borsboom GJJM, Steyerberg EW, Siersema PD. Quality of life after palliative treatment for oesophageal carcinoma - A prospective comparison between stent placement and single dose brachytherapy. *Eur J Cancer*. 2004;40(12):1862-1871. doi:10.1016/j.ejca.2004.04.021

91. Homs MYV, Steyerberg EW, Eijkenboom WMH, et al. Single-dose brachytherapy versus metal stent placement for the palliation of dysphagia from oesophageal cancer: Multicentre randomised trial. *Lancet*. 2004;364(9444):1497-1504. doi:10.1016/S0140-6736(04)17272-3

92. Hoskin P, Rojas A, Fidarova E, et al. IAEA randomised trial of optimal single dose radiotherapy in the treatment of painful bone metastases. *Radiother Oncol*. 2015;116(1):10-14. doi:10.1016/j.radonc.2015.05.008

93. Hoskin PJ, Price P, Easton D, et al. A prospective randomised trial of 4 Gy or 8 Gy single doses in the treatment of metastatic bone pain. *Radiother Oncol*. 1992;23(2):74-78.

94. Howell DD, James JL, Hartsell WF, et al. Single-fraction radiotherapy versus multifraction radiotherapy for palliation of painful vertebral bone metastases - Equivalent efficacy, less toxicity, more convenient: A subset analysis of Radiation Therapy Oncology Group trial 97-14. *Cancer*. 2013;119(4):888-896. doi:10.1002/cncr.27616

95. Huber RM, Fischer R, Hautmann H, et al. Palliative endobronchial brachytherapy for central lung tumors: A prospective, randomized comparison of two fractionation schedules. *Chest*. 1995;107(2):463-470. doi:10.1378/chest.107.2.463

96. Jain S, Kataria T, Bisht SS, et al. Malignant obstructive jaundice - Brachytherapy as a tool for palliation. *J Contemp Brachytherapy*. 2013;5(2):83-88. doi:10.5114/jcb.2013.35563

97. Javed A, Pal S, Dash NR, et al. Palliative stenting with or without radiotherapy for inoperable esophageal carcinoma: a randomized trial. *J Gastrointest Cancer*. 2012;43(1):63-69. doi:10.1007/s12029-010-9206-4

98. Jeremic B, Shibamoto Y, Acimovic L, et al. A randomized trial of three single-dose radiation therapy regimens in the treatment of metastatic bone pain. *Int J Radiat Oncol Biol Phys*. 1998;42(1):161-167. doi:10.1016/s0360-3016(98)00174-6

99. Jeremic B, Fidarova E, Sharma V, et al. The International Atomic Energy Agency (IAEA) randomized trial of palliative treatment of incurable locally advanced non small cell lung cancer (NSCLC) using radiotherapy (RT) and chemotherapy (CHT) in limited resource setting. *Radiother Oncol*. 2015;116(1):21-26. doi:10.1016/j.radonc.2015.06.017

100. Jeremic B, Shibamoto Y, Grujicic D, et al. Short-course radiotherapy in elderly and frail patients with glioblastoma multiforme. A phase II study. *J Neurooncol*. 1999;44(1):85-90. doi:10.1023/A:1006356021734

101. Jeremic B, Shibamoto Y, Milicic B, et al. Short-term chemotherapy and palliative radiotherapy for elderly patients with stage IV non-small cell lung cancer: A phase II study. *Lung Cancer*. 1999;24(1):1-9. doi:10.1016/S0169-5002(99)00011-2

102. Jiang DM, Fyles A, Nguyen LT, et al. Phase i study of local radiation and tremelimumab in patients with inoperable locally recurrent or metastatic breast cancer. *Oncotarget*. 2019;10(31):2947-2958. doi:10.18632/oncotarget.26893

103. Jiao D, Wu G, Ren J, Han X. Study of self-expandable metallic stent placement intraluminal 125I seed strands brachytherapy of malignant biliary obstruction. *Surg Endosc Interv Tech*. 2017;31(12):4996-5005. doi:10.1007/s00464-017-5481-5

104. Jiao D, Zhou X, Li Z, et al. A newly designed biliary brachytherapy drainage catheter for patients with malignant biliary obstruction: A pilot study. *J Cancer Res Ther*. 2020;16(2):286-291. doi:10.4103/jcrt.JCRT_804_19

105. Joensuu H, Eriksson M, Collan J, Balk MH, Leyvraz S, Montemurro M. Radiotherapy for GIST progressing during or after tyrosine kinase inhibitor therapy: A prospective study. *Radiother Oncol*. 2015;116(2):233-238. doi:10.1016/j.radonc.2015.07.025

106. Jóhannsson J, Specht L, Mejer J, Jensen BA. Phase II study of palliative low-dose local radiotherapy in disseminated indolent non-Hodgkin’s lymphoma and chronic lymphocytic leukemia. *Int J Radiat Oncol Biol Phys*. 2002;54(5):1466-1470. doi:10.1016/S0360-3016(02)03050-X

107. Kamstrup MR, Specht L, Skovgaard GL, Gniadecki R. A Prospective, Open-Label Study of Low-Dose Total Skin Electron Beam Therapy in Mycosis Fungoides. *Int J Radiat Oncol Biol Phys*. 2008;71(4):1204-1207. doi:10.1016/j.ijrobp.2007.11.039

108. Kassam Z, Wong RKS, Ringash J, et al. A Phase I/II Study to Evaluate the Toxicity and Efficacy of Accelerated Fractionation Radiotherapy for the Palliation of Dysphagia from Carcinoma of the Oesophagus. *Clin Oncol*. 2008;20(1):53-60. doi:10.1016/j.clon.2007.10.003

109. Kharadi MY, Qadir A, Khan FA, Khuroo MS. Comparative evaluation of therapeutic approaches in stage III and IV squamous cell carcinoma of the thoracic esophagus with conventional radiotherapy and endoscopic treatment in combination and endoscopic treatment alone: A randomized prospective trial. *Int J Radiat Oncol Biol Phys*. 1997;39(2):309-320.

110. Kim BS, Chung HC, Seong JS, Suh CO, Kim GE. Phase II trial for combined external radiotherapy and hyperthermia for unresectable hepatoma. *Cancer Chemother Pharmacol*. 1992;31 Suppl:S119-27. doi:10.1007/bf00687121

111. Komarnicky LT, Phillips TL, Martz K, Asbell S, Isaacson S, Urtasun R. A randomized phase III protocol for the evaluation of misonidazole combined with radiation in the treatment of patients with brain metastases (RTOG-7916). *Int J Radiat Oncol Biol Phys*. 1991;20(1):53-58.

112. Königsrainer A, Riedmann B, De Vries A, et al. Expandable metal stents versus laser combined with radiotherapy for palliation of unresectable esophageal cancer: A prospective randomized trial. *Hepatogastroenterology*. 2000;47(33):724-727.

113. Konski A, Desilvio M, Hartsell W, et al. Continuing evidence for poorer treatment outcomes for single male patients: retreatment data from RTOG 97-14. *Int J Radiat Oncol Biol Phys*. 2006;66(1):229-233. doi:10.1016/j.ijrobp.2006.04.005

114. Kramer GWPM, Gans S, Ullmann E, Van Meerbeeck JP, Legrand CC, Leer JWH. Hypofractionated external beam radiotherapy as retreatment for symptomatic non-small-cell lung carcinoma: An effective treatment? *Int J Radiat Oncol Biol Phys*. 2004;58(5):1388-1393. doi:10.1016/j.ijrobp.2003.09.087

115. Kramer GWPM, Wanders SL, Noordijk EM, et al. Results of the Dutch national study of the palliative effect of irradiation using two different treatment schemes for non-small-cell lung cancer. *J Clin Oncol*. 2005;23(13):2962-2970. doi:10.1200/JCO.2005.01.685

116. Kulhavy M, Sur RK, Levin VC, Donde B, Luhana F. Optimization of single-fraction high dose rate intraluminal brachytherapy in palliation of advanced esophageal cancer: A preliminary report. *EndocurietherapyHyperthermia Oncol*. 1995;11(4):235-239.

117. Kumar A, Sharma A, Mohanti BK, et al. A phase 2 randomized study to compare short course palliative radiotherapy with short course concurrent palliative chemotherapy plus radiotherapy in

© 2022 Fabian A et al. *JAMA Network Open.*

advanced and unresectable head and neck cancer. *Radiother Oncol*. 2015;117(1):145-151. doi:10.1016/j.radonc.2015.07.026

118. Kundel Y, Nasser NJ, Purim O, et al. Phase II Study of Concurrent Capecitabine and External Beam Radiotherapy for Pain Control of Bone Metastases of Breast Cancer Origin. *PLoS ONE*. 2013;8(7). doi:10.1371/journal.pone.0068327

119. Lai YL, Chang HH, Huang MJ, et al. Combined effect of topical arsenic trioxide and radiation therapy on skin-infiltrating lesions of breast cancer - A pilot study. *Anticancer Drugs*. 2003;14(10):825-828. doi:10.1097/00001813-200311000-00008

120. Laltanpui C, Singh YI, Phom YW. Comparison between two different fractionation radiation dose schedules for palliation of advanced solid tumours. *J Med Soc*. 2016;30(1):24-26. doi:10.4103/0972-4958.175796

121. Lange OF, Scheef W, Haase KD, et al. Palliative chemo-radiotherapy with ifosfamide and epirubicin as first-line treatment for high-risk metastatic breast cancer. Results of a prospective multicenter trial. *Cancer Chemother Pharmacol*. 1990;26(SUPPL.):S74-S77. doi:10.1007/BF00685427

122. Langendijk H, De Jong J, Tjwa M, et al. External irradiation versus external irradiation plus endobronchial brachytherapy in inoperable non-small cell lung cancer: A prospective randomized study. *Radiother Oncol*. 2001;58(3):257-268. doi:10.1016/S0167-8140(00)00345-5

123. Lee J, Hodgson D, Chow E, et al. A phase II trial of palliative radiotherapy for metastatic renal cell carcinoma. *Cancer*. 2005;104(9):1894-1900. doi:10.1002/cncr.21410

124. Lehman M, Bernard A, See A, King M, Michael M. A Randomized Phase 3 Trial of Palliative Radiation Therapy Versus Concurrent Chemotherapy and Palliative Radiation Therapy in Patients With Good Performance Status, Locally Advanced, or Metastatic Non-Small Cell Lung Cancer With Symptoms due to Intrathoracic Disease Who are Not Suitable for Radical Chemo-radiation Therapy: Results of the Trans-Tasman Radiation Oncology Group 11.03 Trial. *Pract Radiat Oncol*. Published online 2020. doi:10.1016/j.prro.2020.11.009

125. Li J, Bentzen SM, Renschler M, Mehta MP. Regression after whole-brain radiation therapy for brain metastases correlates with survival and improved neurocognitive function. *J Clin Oncol*. 2007;25(10):1260-1266. doi:10.1200/JCO.2006.09.2536

126. Low DE, Pagliero KM. Prospective randomized clinical trial comparing brachytherapy and laser photoablation for palliation of esophageal cancer. *J Thorac Cardiovasc Surg*. 1992;104(1):173-179. doi:10.1016/s0022-5223(19)34850-0

127. Lupattelli M, Maranzano E, Bellavita R, et al. Short-course palliative radiotherapy in non-small-cell lung cancer: Results of a prospective study. *Am J Clin Oncol*. 2000;23(1):89-93. doi:10.1097/00000421-200002000-00024

128. Macbeth FR, Bolger JJ, Hopwood P, et al. Randomized trial of palliative two-fraction versus more intensive 13-fraction radiotherapy for patients with inoperable non-small cell lung cancer and good performance status. *Clin Oncol*. 1996;8(3):167-175. doi:10.1016/S0936-6555(96)80041-0

129. Majumder D, Chatterjee D, Bandyopadhyay A, Mallick S, Sarkar S, Majumdar A. Single Fraction versus Multiple Fraction Radiotherapy for Palliation of Painful Vertebral Bone Metastases: a Prospective Study. *Indian J Palliat Care*. 2012;18(3):202-206. doi:10.4103/0973-1075.105691

130. Mallick I, Sharma SC, Behera D, Ghoshal S, Oinam AS. Optimization of dose and fractionation of endobronchial brachytherapy with or without external radiation in the palliative management of non-small cell lung cancer: A prospective randomized study. *J Cancer Res Ther*. 2006;2(3):119-125. doi:10.4103/0973-1482.27586

131. Malmström A, Grønberg BH, Marosi C, et al. Temozolomide versus standard 6-week radiotherapy versus hypofractionated radiotherapy in patients older than 60 years with glioblastoma: The Nordic randomised, phase 3 trial. *Lancet Oncol*. 2012;13(9):916-926. doi:10.1016/S1470-2045(12)70265-6

132. Maranzano E, Trippa F, Casale M, et al. 8 Gy single-dose radiotherapy is effective in metastatic spinal cord compression: Results of a phase III randomized multicentre Italian trial. *Radiother Oncol*. 2009;93(2):174-179. doi:10.1016/j.radonc.2009.05.012

133. Marcenaro M, Vagge S, Belgioia L, et al. Ablative or palliative stereotactic body radiotherapy with helical tomotherapy for primary or metastatic lung tumor. *Anticancer Res*. 2013;33(2):655-660.

134. Mattiucci GC, Autorino R, Tringali A, et al. A Phase I study of high-dose-rate intraluminal brachytherapy as palliative treatment in extrahepatic biliary tract cancer. *Brachytherapy*. 2015;14(3):401-404. doi:10.1016/j.brachy.2014.12.002

135. McDermott RL, Armstrong JG, Thirion P, et al. Cancer Trials Ireland (ICORG) 06-34: A multi-centre clinical trial using three-dimensional conformal radiation therapy to reduce the toxicity of palliative radiation for lung cancer. *Radiother Oncol*. 2018;127(2):253-258. doi:10.1016/j.radonc.2018.02.028

136. McGowan DR, Skwarski M, Bradley KM, et al. Buparlisib with thoracic radiotherapy and its effect on tumour hypoxia: A phase I study in patients with advanced non-small cell lung carcinoma. *Eur J Cancer*. 2019;113((McGowan D.R.; Skwarski M.; Campo L.; Gleeson F.V.; Green M.; Maughan T.S.; Muschel R.J.; Prevo R.; Tacconi E.M.C.; Vallis K.A.; McKenna W.G.; Higgins G.S., geoffrey.higgins@oncology.ox.ac.uk) Department of Oncology, University of Oxford, Oxford, United K):87-95. doi:10.1016/j.ejca.2019.03.015

137. Meeuse JJ, Van Der Linden YM, Van Tienhoven G, Gans ROB, Leer JWH, Reyners AKL. Efficacy of radiotherapy for painful bone metastases during the last 12 weeks of life: Results from the dutch bone metastasis study. *Cancer*. 2010;116(11):2716-2725. doi:10.1002/cncr.25062

138. Mehta MP, Rodrigus P, Terhaard CHJ, et al. Survival and Neurologic Outcomes in a Randomized Trial of Motexafin Gadolinium and Whole-Brain Radiation Therapy in Brain Metastases. *J Clin Oncol*. 2003;21(13):2529-2536. doi:10.1200/JCO.2003.12.122

139. Mehta S, Sharma S, Kapoor R, Kochhar R, Mehta V. Quality of life assessment with different radiotherapy schedules in palliative management of advanced carcinoma esophagus: a prospective randomized study. *Indian J Palliat Care*. 2008;14(2):90-96.

140. Meyers CA, Smith JA, Bezjak A, et al. Neurocognitive Function and Progression in Patients With Brain Metastases Treated With Whole-Brain Radiation and Motexafin Gadolinium: Results of a Randomized Phase III Trial. *J Clin Oncol*. 2004;22(1):157-165. doi:10.1200/JCO.2004.05.128

141. Michael M, Wirth A, Ball DL, et al. A phase I trial of high-dose palliative radiotherapy plus concurrent weekly Vinorelbine and Cisplatin in patients with locally advanced and metastatic NSCLC. *Br J Cancer*. 2005;93(6):652-661. doi:10.1038/sj.bjc.6602759

142. Micheletti E, La Face B, Feroldi P, et al. High-dose-rate brachytherapy for poor-prognosis, high grade glioma: (Phase II) preliminary results. *Tumori*. 1996;82(4):339-344.

143. Mohiuddin M, Lingareddy V, Rakinic J, Marks G. Reirradiation for rectal cancer and surgical resection after ultra high doses. *Int J Radiat Oncol Biol Phys*. 1993;27(5):1159-1163.

144. Mudgal A, Arya A, Yadav I, Chaudhary S. Role of hypofractionated palliative radiotherapy in patients with stage four head-and-neck squamous cell carcinoma. *J Cancer Res Ther*. 2019;15(3):528-532. doi:10.4103/jcrt.JCRT_116_18

© 2022 Fabian A et al. *JAMA Network Open.*

145. Muller DA, Wages NA, Wilson DD, et al. STAT RAD: Prospective Dose Escalation Clinical Trial of Single Fraction Scan-Plan-QA-Treat Stereotactic Body Radiation Therapy for Painful Osseous Metastases. *Pract Radiat Oncol*. 2020;10(6):e444-e451. doi:10.1016/j.prro.2020.03.008

146. Mulvenna P, Nankivell M, Barton R, et al. Dexamethasone and supportive care with or without whole brain radiotherapy in treating patients with non-small cell lung cancer with brain metastases unsuitable for resection or stereotactic radiotherapy (QUARTZ): results from a phase 3, non-inferiority, randomised trial. *Lancet*. 2016;388(10055):2004-2014. doi:10.1016/s0140-6736(16)30825-x

147. Murray L, Longo J, Wan J, et al. Phase I dose escalation study of concurrent palliative radiation therapy with sorafenib in three anatomical cohorts (Thorax, Abdomen, Pelvis): The TAP study. *Radiother Oncol*. 2017;124(1):74-79. doi:10.1016/j.radonc.2017.06.007

148. Murthy V, Kumar D, Budrukkar A, Gupta T, Ghosh-Laskar S, Agarwal J. Twice-weekly palliative radiotherapy for locally very advanced head and neck cancers. *Indian J Cancer*. 2016;53(1):138-141. doi:10.4103/0019-509X.180847

149. Murthy V, Thomas K, Foo K, et al. Efficacy of palliative low-dose involved-field radiation therapy in advanced lymphoma: A phase II study. *Clin Lymphoma Myeloma*. 2008;8(4):241-245. doi:10.3816/CLM.2008.n.032

150. Nawrocki S, Krzakowski M, Wasilewska-Tesluk E, et al. Concurrent chemotherapy and short course radiotherapy in patients with stage IIIA to IIIB non-small cell lung cancer not eligible for radical treatment: Results of a randomized phase II study. *J Thorac Oncol*. 2010;5(8):1255-1262. doi:10.1097/JTO.0b013e3181e15d33

151. Nestle U, Nieder C, Abel U, et al. A palliative accelerated irradiation regimen (PAIR) for advanced non-small-cell lung cancer (NSCLC). *Radiother Oncol*. 1996;38(3):195-203. doi:10.1016/0167-8140(96)01706-9

152. Nestle U, Nieder C, Walter K, et al. A palliative accelerated irradiation regimen for advanced non-small-cell lung cancer VS. conventionally fractionated 60 GY: Results of a randomized equivalence study. *Int J Radiat Oncol Biol Phys*. 2000;48(1):95-103. doi:10.1016/S0360-3016(00)00607-6

153. Nielsen OS, Bentzen SM, Sandberg E, Gadeberg CC, Timothy AR. Randomized trial of single dose versus fractionated palliative radiotherapy of bone metastases. *Radiother Oncol*. 1998;47(3):233-240. doi:10.1016/S0167-8140(98)00011-5

154. Omand M, Meredith C. A study of acute side-effects related to palliative radiotherapy treatment of lung cancer. *Eur J Cancer Care (Engl)*. 1994;3(4):149-152.

155. Oosterhof GON, Roberts JT, De Reijke ThM, et al. Strontium89 Chloride versus Palliative Local Field Radiotherapy in Patients with Hormonal Escaped Prostate Cancer: A Phase III Study of the European Organisation for Research and Treatment of Cancer Genitourinary Group. *Eur Urol*. 2003;44(5):519-526. doi:10.1016/S0302-2838(03)00364-6

156. Pal S, Dutta S, Adhikary S, Bhattacharya B, Ghosh B, Patra N. Hemi body irradiation: An economical way of palliation of pain in bone metastasis in advanced cancer. *South Asian J Cancer*. 2014;3(1):28-32. doi:10.4103/2278-330X.126513

157. Pandey KC, Revannasiddaiah S, Pant NK, Nautiyal V, Rastogi M, Gupta MK. Palliative radiotherapy in locally advanced head and neck cancer after failure of induction chemotherapy: comparison of two fractionation schemes. *Indian J Palliat Care*. 2013;19(3):139-145. doi:10.4103/0973-1075.121522

158. Paris KJ, Spanos Jr. WJ, Lindberg RD, Jose B, Albrink F. Phase I-II study of multiple daily fractions for palliation of advanced head and neck malignancies. *Int J Radiat Oncol Biol Phys*. 1993;25(4):657-660.

© 2022 Fabian A et al. *JAMA Network Open.*

159. Penniment MG, De Ieso PB, Harvey JA, et al. Palliative chemoradiotherapy versus radiotherapy alone for dysphagia in advanced oesophageal cancer: a multicentre randomised controlled trial (TROG 03.01). *Lancet Gastroenterol Hepatol*. 2018;3(2):114-124. doi:10.1016/S2468-1253(17)30363-1

160. Picardi V, Deodato F, Guido A, et al. Palliative short-course radiation therapy in rectal cancer: A phase 2 study. *Int J Radiat Oncol Biol Phys*. 2016;95(4):1184-1190. doi:10.1016/j.ijrobp.2016.03.010

161. Porceddu SV, Rosser B, Burmeister BH, et al. Hypofractionated radiotherapy for the palliation of advanced head and neck cancer in patients unsuitable for curative treatment - “Hypo Trial.” *Radiother Oncol*. 2007;85(3):456-462. doi:10.1016/j.radonc.2007.10.020

162. Postow MA, Knox SJ, Goldman DA, et al. A prospective, phase 1 trial of nivolumab, ipilimumab, and radiotherapy in patients with advanced melanoma. *Clin Cancer Res*. 2020;26(13):3193-3201. doi:10.1158/1078-0432.CCR-19-3936

163. Poulter CA, Cosmatos D, Rubin P, et al. A report of RTOG 8206: A phase III study of whether the addition of single dose hemibody irradiation to standard fractionated local field irradiation is more effective than local field irradiation alone in the treatment of symptomatic osseous metastases. *Int J Radiat Oncol Biol Phys*. 1992;23(1):207-214.

164. Prasad NR, Karthigeyan M, Vikram K, Parthasarathy R, Reddy KS. Palliative radiotherapy in esophageal cancer. *Indian J Surg*. 2015;77(1):34-38. doi:10.1007/s12262-013-0817-4

165. Priestman T, Dunn J, Brada M, Rampling R, Baker P. Final results of the Royal College of Radiologists’ trial comparing two different radiotherapy schedules in the treatment of cerebral metastases. *Clin Oncol*. 1996;8(5):308-315. doi:10.1016/s0936-6555(05)80717-4

166. Pugh TJ, Chen C, Rabinovitch R, et al. Phase I trial of bortezomib and concurrent external beam radiation in patients with advanced solid malignancies. *Int J Radiat Oncol Biol Phys*. 2010;78(2):521-526. doi:10.1016/j.ijrobp.2009.07.1715

167. Quilty P, Kirk D, Bolger J, et al. A comparison of the palliative effects of strontium-89 and external beam radiotherapy in metastatic prostate cancer. *Radiother Oncol*. 1994;31(1):33-40. doi:10.1016/0167-8140(94)90411-1

168. Ree AH, Dueland S, Folkvord S, et al. Vorinostat, a histone deacetylase inhibitor, combined with pelvic palliative radiotherapy for gastrointestinal carcinoma: The Pelvic Radiation and Vorinostat (PRAVO) phase 1 study. *Lancet Oncol*. 2010;11(5):459-464. doi:10.1016/S1470-2045(10)70058-9

169. Reed CE, Marsh WH, Carlson LS, Seymore CH, Kratz JM. Prospective, randomized trial of palliative treatment for unresectable cancer of the esophagus. *Ann Thorac Surg*. 1991;51(4):552-556.

170. Rees GJG, Devrell CE, Barley VL, Newman HFV. Palliative radiotherapy for lung cancer: Two versus five fractions. *Clin Oncol*. 1997;9(2):90-95. doi:10.1016/S0936-6555(05)80446-7

171. Rhomberg W, Böhler F, Eiter H, Alton R, Maier R. Radiotherapy and razoxane in the palliative treatment of gastric cancer. *Radiat Oncol Investig*. 1996;4(1):27-32. doi:10.1002/(SICI)1520-6823(1996)4:1<27::AID-ROI5>3.3.CO;2-J

172. Roos DE, O’Brien PC, Smith JG, et al. A role for radiotherapy in neuropathic bone pain: Preliminary response rates from a prospective trial (Trans-Tasman Radiation Oncology Group, TROG 96.05). *Int J Radiat Oncol Biol Phys*. 2000;46(4):975-981. doi:10.1016/S0360-3016(99)00521-0

173. Roos DE, Turner SL, O’Brien PC, et al. Randomized trial of 8Gy in 1 versus 20Gy in 5 fractions of radiotherapy for neuropathic pain due to bone metastases (Trans-Tasman Radiation

© 2022 Fabian A et al. *JAMA Network Open.*

Oncology Group, TROG 96.05). *Radiother Oncol*. 2005;75(1):54-63. doi:10.1016/j.radonc.2004.09.017

174. Rosenblatt E, Jones G, Sur RK, et al. Adding external beam to intra-luminal brachytherapy improves palliation in obstructive squamous cell oesophageal cancer: A prospective multi-centre randomized trial of the International Atomic Energy Agency. *Radiother Oncol*. 2010;97(3):488-494. doi:10.1016/j.radonc.2010.09.001

175. Rudzianskiene M, Inciura A, Gerbutavicius R, et al. Single vs. multiple fraction regimens for palliative radiotherapy treatment of multiple myeloma: A prospective randomised study. *Strahlenther Onkol*. 2017;193(9):742-749. doi:10.1007/s00066-017-1154-5

176. Rudzianskiene M, Inciura A, Juozaityte E, et al. The impact of one fraction of 8 Gy radiotherapy in palliative treatment of multiple myeloma patients with painful bone destructions. *Turk J Med Sci*. 2015;45(2):364-371. doi:10.3906/sag-1403-103

177. Rupinski M, Zagorowicz E, Regula J, et al. Randomized comparison of three palliative regimens including brachytherapy, photodynamic therapy, and APC in patients with malignant dysphagia (CONSORT 1a) (Revised II). *Am J Gastroenterol*. 2011;106(9):1612-1620. doi:10.1038/ajg.2011.178

178. Saha A, Ghosh SK, Roy C, Kayal P. Treatment outcomes in patients with multiple brain metastases: A prospective randomized study. *Clin Cancer Investig J*. 2014;3(4):269-275. doi:10.4103/2278-0513.134467

179. Saikawa Y, Kubota T, Kumagai K, et al. Phase II Study of Chemoradiotherapy With S-1 and Low-Dose Cisplatin for Inoperable Advanced Gastric Cancer. *Int J Radiat Oncol Biol Phys*. 2008;71(1):173-179. doi:10.1016/j.ijrobp.2007.09.010

180. Sakr A, Hashem WB, Ebrahim N, Mashhour KN. Randomized Pilot Study of 20 Gy in 5 Fractions versus 27 Gy in 3 Fractions Radiotherapy for Treating Painful Bone Metastases: A Single Institution Experience. *Asian Pac J Cancer Prev*. 2020;21(6):1807-1811. doi:10.31557/APJCP.2020.21.6.1807

181. Salazar OM, Sandhu T, Da Motta NW, et al. Fractionated half-body irradiation (HBI) for the rapid palliation of widespread, symptomatic, metastatic bone disease: A randomized Phase III trial of the International Atomic Energy Agency (IAEA). *Int J Radiat Oncol Biol Phys*. 2001;50(3):765-775. doi:10.1016/S0360-3016(01)01495-X

182. Sander R, Hagenmueller F, Sander C, Riess G, Classen M. Laser versus laser plus afterloading with iridium-192 in the palliative treatment of malignant stenosis of the esophagus: A prospective, randomized, and controlled study. *Gastrointest Endosc*. 1991;37(4):433-440. doi:10.1016/S0016-5107(91)70775-X

183. Sargeant I, Tobias J, Blackman G, Thorpe S, Glover J, Bown S. Radiotherapy enhances laser palliation of malignant dysphagia: a randomised study. *Gut*. 1997;40(3):362-369. doi:10.1136/gut.40.3.362

184. Sau S, Dutta P, Gayen G, Banerjee S, Basu A. A comparative study of different dose fractionations schedule of thoracic radiotherapy for pain palliation and health-related quality of life in metastatic NSCLC. *Lung India*. 2014;31(4):348-353. doi:10.4103/0970-2113.142111

185. Sayed MM, Abdel-Wanis ME, El-Sayed MI. Single fraction compared with multiple fraction re-irradiations in patients with painful bone metastases. *J Cancer Sci Ther*. 2013;5(2):089-093. doi:10.4172/1948-5956.1000190

186. Sayed M. Different fractionation in whole brain irradiation for multiple brain metastases. *Middle East J Cancer*. 2015;6(2):85-90.

187. Scarantino C, Caplan R, Rotman M, Coughlin C, Demas W, Delrowe J. A phase I/II study to evaluate the effect of fractionated hemibody irradiation in the treatment of osseous metastases--

RTOG 88-22. *Int J Radiat Oncol Biol Phys*. 1996;36(1):37-48. doi:10.1016/s0360-3016(96)00247-7

188. Schäfer U, Micke O, Schüller P, Schuck A, Willich N. The effect of sequential radiochemotherapy in preirradiated malignant gliomas in a phase II study. *J Neurooncol*. 2004;67(1-2):233-239. doi:10.1023/B:NEON.0000021898.31923.bd

189. Schmid EU, Alberts AS, Greeff F, et al. The value of radiotherapy of chemotherapy after intubation for advanced esophageal carcinoma - A prospective randomized trial. *Radiother Oncol*. 1993;28(1):27-30.

190. Schwarzenberger P, Fariss A, Linares L, Nedzi L, Salazar OM. Dose escalation of once weekly oral vinorelbine concurrent with weekly split dose hypofractionated chest radiation for palliation of advanced non-small cell lung cancer: A phase I/II study. *Am J Med Sci*. 2011;341(6):454-459. doi:10.1097/MAJ.0b013e3182127b3f

191. Senkus-Konefka E, Dziadziuszko R, Bednaruk-Młyński E, et al. A prospective, randomised study to compare two palliative radiotherapy schedules for non-small-cell lung cancer (NSCLC). *Br J Cancer*. 2005;92(6):1038-1045. doi:10.1038/sj.bjc.6602477

192. Silva MF, Marta GN, Lisboa FPC, et al. Hypofractionated radiotherapy for complicated bone metastases in patients with poor performance status: a phase II international trial. *Tumori*. 2019;105(2):181-187. doi:10.5301/tj.5000658

193. Singh N, Lakier R, Donde B. Hypofractionated radiation therapy in the treatment of epidemic Kaposi sarcoma--a prospective randomized trial. *Radiother Oncol*. 2008;88(2):211-216. doi:10.1016/j.radonc.2008.03.009

194. Singh V, Kapoor R, Solanki KK, Singh G, Verma GR, Sharma SC. Endoscopic intraluminal brachytherapy and metal stent in malignant hilar biliary obstruction: A pilot study. *Liver Int*. 2007;27(3):347-352. doi:10.1111/j.1478-3231.2006.01439.x

195. Soliman H, Ringash J, Jiang H, et al. Phase II trial of palliative radiotherapy for hepatocellular carcinoma and liver metastases. *J Clin Oncol*. 2013;31(31):3980-3986. doi:10.1200/JCO.2013.49.9202

196. Soni A, Kaushal V, Verma M, Dhull AK, Atri R, Dhankhar R. Comparative evaluation of three palliative radiotherapy schedules in locally advanced head and neck cancer. *World J Oncol*. 2017;8(1):7-14. doi:10.14740/wjon992w

197. Spanos Jr. WJ, Clery M, Perez CA, et al. Late effect of multiple daily fraction palliation schedule for advanced pelvic malignancies (RTOG 8502). *Int J Radiat Oncol Biol Phys*. 1994;29(5):961-967.

198. Spanos Jr. WJ, Perez CA, Marcus S, et al. Effect of rest interval on tumor and normal tissue response - A report of phase III study of accelerated split course palliative radiation for advanced pelvic malignancies (RTOG-8502). *Int J Radiat Oncol Biol Phys*. 1993;25(3):399-403.

199. Spencer GM, Thorpe SM, Blackman GM, et al. Laser augmented by brachytherapy versus laser alone in the palliation of adenocarcinoma of the oesophagus and cardia: A randomised study. *Gut*. 2002;50(2):224-227. doi:10.1136/gut.0500224

200. Spencer GM, Thorpe SM, Sargeant IR, et al. Laser and brachytherapy in the palliation of adenocarcinoma of the oesophagus and cardia. *Gut*. 1996;39(5):726-731. doi:10.1136/gut.39.5.726

201. Spencer K, Velikova G, Henry A, Westhoff P, Hall PT, van der Linden YM. Net Pain Relief After Palliative Radiation Therapy for Painful Bone Metastases: A Useful Measure to Reflect Response Duration? A Further Analysis of the Dutch Bone Metastasis Study. *Int J Radiat Oncol Biol Phys*. 2019;105(3):559-566. doi:10.1016/j.ijrobp.2019.07.009

202. Sprave T, Verma V, Förster R, et al. Bone density and pain response following intensity-modulated radiotherapy versus three-dimensional conformal radiotherapy for vertebral metastases - Secondary results of a randomized trial. *Radiat Oncol*. 2018;13(1). doi:10.1186/s13014-018-1161-4

203. Sprave T, Verma V, Förster R, et al. Local response and pathologic fractures following stereotactic body radiotherapy versus three-dimensional conformal radiotherapy for spinal metastases - a randomized controlled trial. *BMC Cancer*. 2018;18(1). doi:10.1186/s12885-018-4777-8

204. Sprave T, Verma V, Forster R, et al. Quality of life and radiation-induced late toxicity following intensity-modulated versus three-dimensional conformal radiotherapy for patients with spinal bone metastases: Results of a randomized trial. *Anticancer Res*. 2018;38(8):4953-4960. doi:10.21873/anticanres.12813

205. Sprave T, Verma V, Förster R, et al. Quality of life following stereotactic body radiotherapy versus three-dimensional conformal radiotherapy for vertebral metastases: Secondary analysis of an exploratory Phase II randomized trial. *Anticancer Res*. 2018;38(8):4961-4968. doi:10.21873/anticanres.12814

206. Sprave T, Verma V, Förster R, et al. Radiation-induced acute toxicities after image-guided intensity-modulated radiotherapy versus three-dimensional conformal radiotherapy for patients with spinal metastases (IRON-1 trial): First results of a randomized controlled trial. *Strahlenther Onkol*. 2018;194(10):911-920. doi:10.1007/s00066-018-1333-z

207. Sprave T, Verma V, Förster R, et al. Randomized phase II trial evaluating pain response in patients with spinal metastases following stereotactic body radiotherapy versus three-dimensional conformal radiotherapy. *Radiother Oncol*. 2018;128(2):274-282. doi:10.1016/j.radonc.2018.04.030

208. Steenland E, Leer J, Van Houwelingen H, et al. The effect of a single fraction compared to multiple fractions on painful bone metastases: A global analysis of the Dutch Bone Metastasis Study. *Radiother Oncol*. 1999;52(2):101-109. doi:10.1016/S0167-8140(99)00110-3

209. Stout R, Barber P, Burt P, et al. Clinical and quality of life outcomes in the first United Kingdom randomized trial of endobronchial brachytherapy (intraluminal radiotherapy) vs. external beam radiotherapy in the palliative treatment of inoperable non- small cell lung cancer. *Radiother Oncol*. 2000;56(3):323-327. doi:10.1016/S0167-8140(00)00252-8

210. Strøm HH, Bremnes RM, Sundstrøm SH, Helbekkmo N, Fløtten ., Aasebø U. Concurrent palliative chemoradiation leads to survival and quality of life benefits in poor prognosis stage III non-small-cell lung cancer: A randomised trial by the Norwegian Lung Cancer Study Group. *Br J Cancer*. 2013;109(6):1467-1475. doi:10.1038/bjc.2013.466

211. Strøm HH, Bremnes RM, Sundstrøm SH, Helbekkmo N, Aasebø U. Poor prognosis patients with inoperable locally advanced NSCLC and large tumors benefit from palliative chemoradiotherapy: A subset analysis from a randomized clinical phase III trial. *J Thorac Oncol*. 2014;9(6):825-833. doi:10.1097/JTO.0000000000000184

212. Strøm HH, Bremnes RM, Sundstrøm SH, Helbekkmo N, Aasebø U. How do elderly poor prognosis patients tolerate palliative concurrent chemoradiotherapy for locally advanced non-small-cell lung cancer stage III? A subset analysis from a clinical phase III trial. *Clin Lung Cancer*. 2015;16(3):183-192. doi:10.1016/j.cllc.2014.08.005

213. Sundstrøm S, Bremnes R, Brunsvig P, et al. Immediate or delayed radiotherapy in advanced non-small cell lung cancer (NSCLC)? Data from a prospective randomised study. *Radiother Oncol*. 2005;75(2):141-148. doi:10.1016/j.radonc.2005.03.028

214. Sundstrøm S, Bremnes R, Aasebo U, et al. Hypofractionated palliative radiotherapy (17 Gy per two fractions) in advanced non-small-cell lung carcinoma is comparable to standard fractionation

© 2022 Fabian A et al. *JAMA Network Open.*

for symptom control and survival: A national phase III trial. *J Clin Oncol*. 2004;22(5):801-810. doi:10.1200/JCO.2004.06.123

215. Sundstrøm S, Bremnes RM, Brunsvig P, Aasebø U, Kaasa S. Palliative thoracic radiotherapy in locally advanced non-small cell lung cancer: Can quality-of-life assessments help in selection of patients for short- or long-course radiotherapy? *J Thorac Oncol*. 2006;1(8):816-824.

216. Sur R, Donde B, Falkson C, et al. Randomized prospective study comparing high-dose-rate intraluminal brachytherapy (HDRILBT) alone with HDRILBT and external beam radiotherapy in the palliation of advanced esophageal cancer. *Brachytherapy*. 2004;3(4):191-195. doi:10.1016/j.brachy.2004.09.004

217. Sur RK, Donde B, Levin V, Mannell A, Merwe DV. Chemosensitization and brachytherapy in palliation of advanced esophageal cancer. *J Brachytherapy Int*. 1999;15(3-4):177-187.

218. Sur RK, Levin CV, Donde B, Sharma V, Miszczyk L, Nag S. Prospective randomized trial of HDR brachytherapy as a sole modality in palliation of advanced esophageal carcinoma - An International Atomic Energy Agency study. *Int J Radiat Oncol Biol Phys*. 2002;53(1):127-133. doi:10.1016/S0360-3016(02)02702-5

219. Sur R, Donde B, Levin V, Mannell A. Fractionated high dose rate intraluminal brachytherapy in palliation of advanced esophageal cancer. *Int J Radiat Oncol Biol Phys*. 1998;40(2):447-453. doi:10.1016/s0360-3016(97)00710-4

220. Swaminath A, Wright JR, Tsakiridis TK, et al. A Phase II Trial of Erlotinib and Concurrent Palliative Thoracic Radiation for Patients with Non-Small-Cell Lung Cancer. *Clin Lung Cancer*. 2016;17(2):142-149. doi:10.1016/j.cllc.2015.09.008

221. Takhar HS, Singhal N, Gowda R, Penniment M, Takhar P, Brown MP. Phase i study evaluating the safety and efficacy of oral panobinostat in combination with radiotherapy or chemoradiotherapy in patients with inoperable stage III non-small-cell lung cancer. *Anticancer Drugs*. 2015;26(10):1069-1077. doi:10.1097/CAD.0000000000000282

222. Tey J, Zheng H, Soon YY, et al. Palliative radiotherapy in symptomatic locally advanced gastric cancer: A phase II trial. *Cancer Med*. 2019;8(4):1447-1458. doi:10.1002/cam4.2021

223. Thomas R. Hypofractionated radiotherapy as palliative treatment in poor prognosis patients with high grade glioma. *Radiother Oncol*. 1994;33(2):113-116. doi:10.1016/0167-8140(94)90064-7

224. Tian Q, Zhang F, Wang Y. Clinical assessment of palliative radiotherapy for pancreatic cancer. *Cancer Radiother*. 2018;22(8):778-783. doi:10.1016/j.canrad.2018.02.004

225. Tortochaux J, Tao Y, Tournay E, et al. Randomized phase III trial (GORTEC 98-03) comparing re-irradiation plus chemotherapy versus methotrexate in patients with recurrent or a second primary head and neck squamous cell carcinoma, treated with a palliative intent. *Radiother Oncol*. 2011;100(1):70-75. doi:10.1016/j.radonc.2011.06.025

226. Trovo M, Revelant A, Scd J, et al. Radical Hemithoracic Radiotherapy vs. Palliative Radiotherapy in Non-metastatic Malignant Pleural Mesothelioma: results from a Phase III, Randomized Clinical Trial. *Int J Radiat Oncol Biol Phys*. Published online 2020. doi:10.1016/j.ijrobp.2020.11.057

227. Tyc-Szczepaniak D, Wyrwicz L, Kepka L, et al. Palliative radiotherapy and chemotherapy instead of surgery in symptomatic rectal cancer with synchronous unresectable metastases: A phase II study. *Ann Oncol*. 2013;24(11):2829-2834. doi:10.1093/annonc/mdt363

228. Uppelschoten JM. Single-dose radiotherapy (6 Gy): Palliation in painful bone metastases. *Radiother Oncol*. 1995;36(3):198-202. doi:10.1016/0167-8140(95)01587-7

229. Urba SG, Turrisi III AT. Split-course accelerated radiation therapy combined with carboplatin and 5-fluorouracil for palliation of metastatic or unresectable carcinoma of the esophagus. *Cancer*.

1995;75(2):435-439. doi:10.1002/1097-0142(19950115)75:2<435::AID-CNCR2820750204>3.0.CO;2-T

230. van der Linden YM, Steenland E, van Houwelingen HC, et al. Patients with a favourable prognosis are equally palliated with single and multiple fraction radiotherapy: Results on survival in the Dutch Bone Metastasis Study. *Radiother Oncol*. 2006;78(3):245-253. doi:10.1016/j.radonc.2006.02.007

231. van der Linden Y, Dijkstra S, Vonk E, Marijnen C, Leer J. Prediction of survival in patients with metastases in the spinal column: results based on a randomized trial of radiotherapy. *Cancer*. 2005;103(2):320-328. doi:10.1002/cncr.20756

232. van der Linden Y, Lok J, Steenland E, et al. Single fraction radiotherapy is efficacious: a further analysis of the Dutch Bone Metastasis Study controlling for the influence of retreatment. *Int J Radiat Oncol Biol Phys*. 2004;59(2):528-537. doi:10.1016/j.ijrobp.2003.10.006

233. Veluthattil AC, Sudha SP, Kandasamy S, Chakkalakkoombil SV. Effect of Hypofractionated, Palliative Radiotherapy on Quality of Life in Late-Stage Oral Cavity Cancer: A Prospective Clinical Trial. *Indian J Palliat Care*. 2019;25(3):383-390. doi:10.4103/ijpc.Ijpc_115_18

234. Vokes EE, Moormeier JA, Ratain MJ, et al. 5-Fluorouracil, leucovorin, hydroxyurea, and escalating doses of continuous-infusion cisplatin with concomitant radiotherapy: A clinical and pharmacologic study. *Cancer Chemother Pharmacol*. 1992;29(3):178-184.

235. Weppelmann B, Wheeler RH, Peters GE, et al. Treatment of recurrent head and neck cancer with 5-fluorouracil, hydroxyurea, and reirradiation. *Int J Radiat Oncol Biol Phys*. 1992;22(5):1051-1056.

236. Westhoff PG, de Graeff A, Monninkhof EM, et al. Quality of Life in Relation to Pain Response to Radiation Therapy for Painful Bone Metastases. *Int J Radiat Oncol Biol Phys*. 2015;93(3):694-701. doi:10.1016/j.ijrobp.2015.06.024

237. Westhoff PG, Verdam MGE, Oort FJ, et al. Course of Quality of Life After Radiation Therapy for Painful Bone Metastases: A Detailed Analysis From the Dutch Bone Metastasis Study. *Int J Radiat Oncol Biol Phys*. 2016;95(5):1391-1398. doi:10.1016/j.ijrobp.2016.03.032

238. Wiegel T, Bottke D, Kreusel KM, et al. External beam radiotherapy of choroidal metastases - Final results of a prospective study of the German Cancer Society (ARO 95-08). *Radiother Oncol*. 2002;64(1):13-18. doi:10.1016/S0167-8140(02)00134-2

239. Wieners G, Pech M, Rudzinska M, et al. CT-guided interstitial brachytherapy in the local treatment of extrahepatic, extrapulmonary secondary malignancies. *Eur Radiol*. 2006;16(11):2586-2593. doi:10.1007/s00330-006-0241-2

240. Wong KH, Gaffney C, Chow SM, Chan TM. Results of a phase II clinical trial of 2-fractionated half-body irradiation in treatment of patients with multiple painful bony metastases. *J Hong Kong Coll Radiol*. 2005;8(3):162-171.

241. Xiang Z, Mo Z, Li G, et al. 125I brachytherapy in the palliation of painful bone metastases from lung cancer after failure or rejection of conventional treatments. *Oncotarget*. 2016;7(14):18384-18393. doi:10.18632/oncotarget.7584

242. Yadav BS, Kapoor R, Sharma SC, Kochhar R, Patel FD. Radiation schedules for palliation in carcinoma esophagus. *JK Sci*. 2007;9(3):127-131.

243. Yarnold JR. 8 Gy single fraction radiotherapy for the treatment of metastatic skeletal pain: Randomised comparison with a multifraction schedule over 12 months of patient follow-up. *Radiother Oncol*. 1999;52(2):111-121. doi:10.1016/S0167-8140(99)00097-3

244. Yoshikawa T, Tsuburaya A, Hirabayashi N, et al. A phase I study of palliative chemoradiation therapy with paclitaxel and cisplatin for local symptoms due to an unresectable primary

advanced or locally recurrent gastric adenocarcinoma. *Cancer Chemother Pharmacol*. 2009;64(6):1071-1077. doi:10.1007/s00280-009-0963-3

245. Zamagni A, Buwenge M, Macchia G, et al. Accelerated middle half body radiotherapy in bone metastases from prostate cancer: A phase I study (SHARON project). *Anticancer Res*. 2019;39(9):5065-5069. doi:10.21873/anticanres.13699

246. Zhao F, Tian W, Zeng M, et al. Apatinib alone or combined with radiotherapy in metastatic prostate cancer: Results from a pilot, multicenter study. *Oncotarget*. 2017;8(67):110774-110784. doi:10.18632/oncotarget.22719

247. Zhu HD, Guo JH, Huang M, et al. Irradiation stents vs. conventional metal stents for unresectable malignant biliary obstruction: A multicenter trial. *J Hepatol*. 2018;68(5):970-977. doi:10.1016/j.jhep.2017.12.028

248. Zhu HD, Guo JH, Mao AW, et al. Conventional stents versus stents loaded with 125iodine seeds for the treatment of unresectable oesophageal cancer: A multicentre, randomised phase 3 trial. *Lancet Oncol*. 2014;15(6):612-619. doi:10.1016/S1470-2045(14)70131-7

249. Zimmermann FB, Jeremic B, Lersch C, Geinitz H, Hennig M, Molls M. Dose escalation of concurrent hypofractionated radiotherapy and continuous infusion 5-FU-chemotherapy in advanced adenocarcinoma of the pancreas. *Hepatogastroenterology*. 2005;52(61):246-250.

250. Zygogianni A, Platoni K, Patriki E, et al. A randomized study comparing two hypofractioned 3-D conformal radiotherapy for stage IIIb-IV non small cell lung cancer. *J BUON*. 2020;25(2):842-847.

List of included ongoing trials of palliative radiotherapy registered on “ClinicalTrials.gov”.

1. AHS Cancer Control Alberta. Chemoradiotherapy for Advanced Esophageal Cancer. *NCT02297217*. Published online 2019.

2. AHS Cancer Control Alberta, Cross Cancer Institute. Trial of Combined Radiotherapy and Vertebroplasty for Patients With Painful Metastatic Spinal Lesions. *NCT04242589*. Published online 2020.

3. Azienda Ospedaliera Universitaria di Bologna Policlinico S. Orsola Malpighi. Short Course Radiation Therapy in Palliative Treatment of Abdominal Cancer. *NCT03775005*. Published online 2017.

4. Azienda Ospedaliera Universitaria di Bologna Policlinico S. Orsola Malpighi. Short Course Radiation Therapy in Palliative Treatment of Brain Metastases. *NCT03525301*. Published online 2017.

5. Azienda Ospedaliera Universitaria di Bologna Policlinico S. Orsola Malpighi. Short Course Radiation Therapy in Palliative Treatment of Complicated Bone Metastases. *NCT03503682*. Published online 2017.

6. Azienda Ospedaliera Universitaria di Bologna Policlinico S. Orsola Malpighi. Short Course Radiation Therapy in Palliative Treatment of Esophageal Cancer. *NCT03804203*. Published online 2017.

7. Azienda Ospedaliera Universitaria di Bologna Policlinico S. Orsola Malpighi. Short Course Radiation Therapy in Palliative Treatment of Head and Neck Cancer. *NCT03804073*. Published online 2017.

8. Azienda Ospedaliera Universitaria di Bologna Policlinico S. Orsola Malpighi. Short Course Radiation Therapy in Palliative Treatment of Pelvic Cancer. *NCT03804333*. Published online 2017.

9. Azienda Ospedaliera Universitaria di Bologna Policlinico S. Orsola Malpighi. Short Course Radiation Therapy in Palliative Treatment of Thoracic Cancer. *NCT03804307*. Published online 2017.

10. Baptist Health South Florida, Medtronic. Radiation Therapy Alone Versus Radiation Therapy Plus Radiofrequency Ablation (RFA)/Vertebral Augmentation. *NCT04375891*. Published online 2020.

11. British Columbia Cancer Agency. SUPR-3D: Simple Unplanned Palliative Radiotherapy Versus 3D Conformal Radiotherapy for Patients With Bone Metastases. *NCT03694015*. Published online 2019.

12. Canadian Cancer Trials Group. Study of Palliative Radiotherapy for Symptomatic Hepatocellular Carcinoma and Liver Metastases. *NCT02511522*. Published online 2015.

13. Canadian Cancer Trials Group, Trans Tasman Radiation Oncology Group. Study Comparing Stereotactic Body Radiotherapy vs Conventional Palliative Radiotherapy (CRT) for Spinal Metastases. *NCT02512965*. Published online 2015.

14. Care Partners, OHSU Knight Cancer Institute, Oregon Health, Science University. Access to Single-Fraction Palliative Radiation Therapy in Cancer Patients Enrolled in Hospice. *NCT04049188*. Published online 2020.

15. Chang Gung Memorial Hospital. Neurocognitive Impact and Dose-Effect Relationship of Hippocampal Avoidance During Whole Brain Radiotherapy Plus Simultaneous Integrated Boost - A Prospective Follow-up Study. *NCT03223675*. Published online 2016.

16. City of Hope Medical Center, National Cancer Institute. Pembrolizumab and Palliative Radiation Therapy in Treating Patients With Metastatic Esophagus, Stomach, or Gastroesophageal Junction Cancer. *NCT02830594*. Published online 2016.

17. Di Deng, Wuhan University. Efficacy of Palliative Low-Dose Involved-Field Radiation Therapy for Recurrent Advanced Follicular Lymphoma. *NCT02438501*. Published online 2015.

18. EMD Serono Research, Development Institute Inc, Merck KGaA D Germany, EMD Serono. Phase 1 Trial of MSC2490484A, an Inhibitor of a DNA-dependent Protein Kinase, in Combination With Radiotherapy. *NCT02516813*. Published online 2015.

19. EMD Serono Research, Development Institute Inc, Merck KGaA D Germany, EMD Serono. Study of Avelumab-M3814 Combinations. *NCT03724890*. Published online 2018.

20. Emory University, Pfizer. Radiation Therapy, Palbociclib, and Hormone Therapy in Treating Breast Cancer Patients With Bone Metastasis. *NCT03691493*. Published online 2019.

21. Juergen Debus, Heidelberg University, University Hospital Heidelberg. Whole Brain Radiation Therapy Alone vs. Radiosurgery for SCLC Patients With 1-10 Brain Metastases. *NCT03297788*. Published online 2017.

22. Lawson Health Research Institute. Standard Versus Radiobiologically-Guided Dose Selected SBRT in Liver Cancer. *NCT04745390*. Published online 2021.

23. Lipomedix Pharmaceuticals Inc. Intravenously Administered Liposomal PROMITIL in Combination With External Beam Radiotherapy in Cancer Patients. *NCT03823989*. Published online 2019.

24. Ludwig Institute for Cancer Research, Bristol-Myers Squibb, Conquer Cancer Foundation. Pilot Study of the Safety/Efficacy of Combination Checkpoint Blockade + External Beam Radiotherapy in Stage IV Melanoma. *NCT02659540*. Published online 2016.

© 2022 Fabian A et al. *JAMA Network Open.*

25. M.D. Anderson Cancer Center, National Cancer Institute. Single Fraction or Multi-fraction Palliative Radiation Therapy for the Improvement of Quality of Life in Patients With Metastatic Gynecologic Cancers. *NCT04516135*. Published online 2020.

26. Maastricht Radiation Oncology, The Netherlands Cancer Institute, Maastricht University Medical Center, Zuyderland Medical Centre. Re-Induction After Initial Response With Immune Therapy With Radiotherapy in Lung Cancer. *NCT03406468*. Published online 2019.

27. Mary Feng M, AstraZeneca, University of California SF. Hypofractionated Radiotherapy Followed by Durvalumab With or Without Tremelimumab for the Treatment of Liver Cancer After Progression on Prior PD-1 Inhibition. *NCT04430452*. Published online 2021.

28. Megan Daly M, Merck Sharp, Dohme Corp., University of California D. UCDCC#272: IL-2, Radiotherapy, and Pembrolizumab in Patients Refractory to Checkpoint Blockade. *NCT03474497*. Published online 2019.

29. Melanoma, Skin Cancer Trials Limited, Melanoma Institute Australia. Radiotherapy & Combi in Metastatic Melanoma. *NCT02392871*. Published online 2015.

30. Memorial Sloan Kettering Cancer Center. Proton Re-Irradiation for Recurrent Head and Neck Cancer. *NCT03217188*. Published online 2017.

31. Memorial Sloan Kettering Cancer Center. Study of Palliative Radiation Therapy vs. no Palliative Radiation Therapy for Patients With High Risk Bone Metastases That Are Not Causing Significant Pain. *NCT03523351*. Published online 2018.

32. Memorial Sloan Kettering Cancer Center. Radiation Therapy to Relieve Symptoms in Patients With Advanced Non-Small Cell Lung Cancer (NSCLC). *NCT04384146*. Published online 2020.

33. Memorial Sloan Kettering Cancer Center, Cedars-Sinai Medical Center. Homologous Recombination Repair Status as a Biomarker of Response in Locally Recurrent/Metastatic Triple Negative Breast Cancer Patients Treated With Concurrent Cisplatin and Radiation Therapy. *NCT02422498*. Published online 2015.

34. Memorial Sloan Kettering Cancer Center, Merck Sharp, Dohme Corp., Cedars-Sinai Medical Center. Study to Assess the Efficacy of Pembrolizumab Plus Radiotherapy in Metastatic Triple Negative Breast Cancer Patients. *NCT02730130*. Published online 2016.

35. National Cancer Institute. Ropidoxuridine in Treating Patients With Advanced Gastrointestinal Cancer Undergoing Radiation Therapy. *NCT02381561*. Published online 2016.

36. National Taiwan University Hospital. The Ave-CRT Study for Newly Diagnosed Metastatic Esophageal Squamous Cell Carcinoma. *NCT03800953*. Published online 2019.

37. Oncology Institute of Southern Switzerland, Ente Ospedaliero Cantonale B, Istituto Cantonale di Patologia, Clinical Trial Unit Ente Ospedaliero Cantonale. Atezolizumab Plus 8 Gy Single-fraction Radiotherapy for Advanced Oligoprogressive NSCLC. *NCT04549428*. Published online 2020.

38. Oslo University Hospital, Bristol-Myers Squibb. Safety and Feasibility of Irradiation and Nivolumab in Esophageal Cancer (INEC). *NCT03544736*. Published online 2018.

39. Roswell Park Cancer Institute. Single-Fraction SBRT Versus Standard Palliative Radiation Therapy in Treating Patients With Metastatic Cancer. *NCT04068649*. Published online 2019.

40. Royal Marsden NHS Foundation Trust, AstraZeneca, Cancer Research UK, RM/ICR Biomedical Research Centre. Phase I Study to Assess Safety of AZD6738 Alone and in Combination With Radiotherapy in Patients With Solid Tumours. *NCT02223923*. Published online 2014.

41. Royal Marsden NHS Foundation Trust, Merck Sharp, Dohme Corp., Institute of Cancer Research UK, National Institute for Health Research UK. Pembrolizumab and Palliative Radiotherapy in Lung. *NCT02587455*. Published online 2016.

42. Shanghai Chest Hospital, The First Affiliated Hospital of Suzhou University. KN046 in Patients With Recurrent or Metastatic Esophageal Squamous Cell Carcinoma. *NCT03927495*. Published online 2019.

43. Sidney Kimmel Comprehensive Cancer Center at Johns Hopkins. Palliative Stereotactic Radiation for Pancreatic or Periampullary Adenocarcinoma. *NCT01781728*. Published online 2013.

44. Sir Mortimer B. Davis - Jewish General Hospital. GRID Therapy as Palliative Radiation for Patients With Advanced and Symptomatic Tumors. *NCT02333110*. Published online 2015.

45. Stanford University. Palliative RT & Anti-PD-1/PD-L1 Checkpoint Blockade in Metastatic Merkel Cell Carcinoma. *NCT03988647*. Published online 2019.

46. Sunnybrook Health Sciences Centre, Focused Ultrasound Foundation. MRG FU With Radiotherapy for Palliation of H&N Cancer. *NCT03218475*. Published online 2016.

47. Tata Medical Center. Palliative Hypofractionated Radiotherapy in Advanced Incurable Breast Cancer. *NCT03511781*. Published online 2015.

48. Tata Medical Center. Hypo-Fractionated Radiotherapy in Breast Cancer. *NCT03669497*. Published online 2018.

49. Tata Memorial Hospital, Tata Memorial Centre. Palliative Radiation for Advanced Cervical Cancer. *NCT03997110*. Published online 2020.

50. Theodore S. Johnson, National Cancer Institute, Augusta University, Emory University. Pediatric Trial of Indoximod With Chemotherapy and Radiation for Relapsed Brain Tumors or Newly Diagnosed DIPG. *NCT04049669*. Published online 2019.

51. Turku University Hospital. MR Imaging- Guided High Intensity Focused Ultrasound (HIFU) Therapy of Bone Metastases. *NCT03106675*. Published online 2017.

52. UMC Utrecht, Turku University Hospital, Universitätsklinikum Köln, et al. Focused Ultrasound and RadioTHERapy for Noninvasive Palliative Pain Treatment in Patients With Bone Metastases. *NCT04307914*. Published online 2020.

53. University of Aarhus. PAlliative RAdioTherapy to Lung Cancer A Randomized Multicentre Phase III Study. *NCT03632603*. Published online 2018.

54. University of Cincinnati, Children's Hospital Medical Center C. Hypofractionated Radiotherapy for Recurrent DIPG. *NCT03841435*. Published online 2018.

55. University of Oklahoma. "QUAD SHOT" Radiotherapy With Pembrolizumab in Patients With Recurrent Head & Neck Cancer. *NCT04373642*. Published online 2020.

56. University of Oxford, Merck KGaA D Germany. M6620 Plus Standard Treatment in Oesophageal and Other Cancer. *NCT03641547*. Published online 2018.

57. University of Southern California, National Cancer Institute. Low-Dose Radiotherapy in Treating Painful Bone Metastases in Patients With Multiple Myeloma. *NCT03858205*. Published online 2019.

58. University of Texas Southwestern Medical Center. "Re-Priming" RT After Incomplete Response to CAR-T in R/R NHL. *NCT04601831*. Published online 2020.

© 2022 Fabian A et al. *JAMA Network Open.*

59. University of Washington, National Cancer Institute, AstraZeneca. Durvalumab, Tremelimumab and Hypofractionated Radiation Therapy in Treating Patients With Recurrent or Metastatic Head and Neck Squamous Cell Carcinoma. *NCT03522584*. Published online 2018.

60. University of Wisconsin M. Microwave Ablation With MRI-Guided SBRT Boost in Renal Cell Carcinoma. *NCT02782715*. Published online 2018.

61. University of Wisconsin M, National Cancer Institute, Bristol-Myers Squibb, Apeiron Biologics, Provenance Biopharmaceuticals. IT-hu14.18-IL2 With Radiation, Nivolumab and Ipilimumab for Melanoma. *NCT03958383*. Published online 2020.

62. Varian Medical. Feasibility Study of FLASH Radiotherapy for the Treatment of Symptomatic Bone Metastases. *NCT04592887*. Published online 2020.

63. Wake Forest University Health Sciences, National Cancer Institute. Palliative Radiation Therapy in Reducing Pain in Patients With Bone Metastasis. *NCT02699697*. Published online 2016.

64. Wake Forest University Health Sciences, National Cancer Institute. Quad Shot Radiotherapy in Combination With Immune Checkpoint Inhibition. *NCT04454489*. Published online 2021.

65. Washington University School of Medicine, Goldman Sachs Foundation. Palliative Lattice Stereotactic Body Radiotherapy (SBRT) for Patients With Sarcoma, Thoracic, Abdominal, and Pelvic Cancers. *NCT04553471*. Published online 2020.

66. Wuhan University. The Palliative Benefit of Involved-site Radiotherapy for Patients With Advanced-stage Diffuse Large B-cell Lymphoma. *NCT02449278*. Published online 2015.

67. Yang Yang, The Affiliated Nanjing Drum Tower Hospital of Nanjing University Medical School. Pulsed Low Dose Rate Radiation Therapy for Gastric Cancer Patients With Peritoneal Metastasis. *NCT03061162*. Published online 2017.

© 2022 Fabian A et al. *JAMA Network Open.*
